# Tracing the sources of suspended sediment and particle-bound trace metal elements in an urban catchment coupling elemental and isotopic geochemistry, and fallout radionuclides

**DOI:** 10.1007/s11356-018-2892-3

**Published:** 2018-08-10

**Authors:** Claire Froger, Sophie Ayrault, Olivier Evrard, Gaël Monvoisin, Louise Bordier, Irène Lefèvre, Cécile Quantin

**Affiliations:** 1 Laboratoire des Sciences du Climat et de l’Environnement (LSCE/IPSL), CEA-CNRS-UVSQ, Université Paris-Saclay, Gif-sur-Yvette, France; 2 Géosciences Paris Sud (GEOPS), Université Paris-Sud, Université Paris-Saclay, Orsay, France

**Keywords:** Road deposited sediment, Lead isotopes, Sediment fingerprinting, Recent sediment, Legacy contamination

## Abstract

The excessive supply of contaminants from urban areas to rivers during the last centuries has led to deleterious impacts on aquatic ecosystems. The sources, the behavior, and the dynamics of these contaminants must be better understood in order to reduce this excessive anthropogenic pollution. Accordingly, the current research investigated the particle-bound trace element (TE) contamination of the 900-km^2^ Orge River (Seine basin, France) and the potential sources of these particles (agricultural or forest soils, channel banks, road deposited sediments), through the analysis of multiple fallout radionuclides, elemental geochemistry, and lead isotopic composition on suspended particulate matter (SPM) collected during a hydrological year at four stations following an increasing urbanization gradient (300 to 5000 inhab.km^−2^). Fallout radionuclide measurements showed an increasing contribution of recently eroded particles from urban areas to the SPM in downstream direction. However, this contribution varied depending on hydrological conditions. A greater contribution of particles originating from urban areas was observed during low stage periods. On the contrary, the contribution of agricultural soils and channel banks that are less enriched in contaminants and fallout radionuclides was higher during seasonal floods, which explained the dilution of radionuclide contents in sediment transiting the river during those events. Trace element contamination of SPM in Cu, Zn, Pb, and Sb increased from moderate to significant levels with urban pressure in downstream direction (with corresponding enrichment factors raising from 2 to 6). In addition, Pb isotopic ratios indicated that the main source of Pb corresponded to the “urban” signature found in road deposited sediments. The low variations in lead isotope ratios found in the SPM for contrasting hydrological conditions demonstrated the occurrence of a single source of Pb contamination. These results demonstrate the need to better manage urban runoff during both flood and low precipitation events to prevent the supply of diffuse particle-bound contamination to rivers draining urban areas.

**Electronic supplementary material:**

The online version of this article (10.1007/s11356-018-2892-3) contains supplementary material, which is available to authorized users.

## Introduction

Human activities have strongly disturbed river ecosystems across the world through the significant supply of contaminants including an excess of nutrients and trace elements (TE) to the rivers (Meybeck 2001; Vörösmarty et al. 2010). When these inputs exceed a certain level, a better management of river pollution is required through the reduction of industrial and domestic discharges or the improvement of wastewater treatment (Middelkoop 2000; Rabalais et al. 1996; Santschi et al. 2001). The Seine River Basin draining Paris Megacity provides an emblematic example of anthropogenic river basin that has been exposed to significant pollution levels for more than one century (Grosbois et al. 2006; Meybeck et al. 2016).

The temporal evolution of contamination levels in the Seine River basin was previously reconstructed through the calculation of pollutant budgets and fluxes, in particular for metals such as Cu, Zn, Cd, and Pb (Thévenot et al. 2007). Another strategy to reconstruct the historical contamination of the basin consists in analyzing sediment cores collected in frequently flooded areas in alluvial plains. These investigations showed that particulate TE contamination in the Seine River reached a peak in the 1960s when the industrial development in the basin was maximal (Ayrault et al. 2012; Le Cloarec et al. 2011; Lorgeoux et al. 2016). Since 1980, a global decrease in TE contamination levels has been observed in sediment transiting the Seine River as a result of the deindustrialization of the region and the adoption of environmental protection laws (Meybeck et al. 2016). For instance, the French law on water resources in 1992 and the European Water Framework Directive in 2000 contributed to define target quality requirements at the catchment scale. However, despite these efforts, particulate-bound TE concentrations remain at a high level nowadays (Horowitz et al. 1999; Le Pape et al. 2012; Priadi et al. 2011b; Thévenot et al. 2007).

Accordingly, the current sources and pathways supplying particulate pollutants to the rivers draining the Seine basin should be identified. Their respective contribution should be quantified to properly guide management decisions to improve the water and sediment quality. To this end, sediment tracing or fingerprinting techniques have been developed to identify the sources supplying material to the river through the analysis of conservative physico-chemical properties in both sources and sediment (Haddadchi et al. 2013). However, these methods have mostly been developed and applied in agricultural and rural catchments (Foucher et al. 2015; Le Gall et al. 2017b; Wilkinson et al. 2013). The identification of sediment sources in an urban basin as complex as the Seine River basin requires the inclusion of multiple specific sources of particles and contamination such as waste water treatment plants and urban runoff.

The identification of contamination sources using geochemical tracers including TE concentrations associated with their isotopic composition has been widely used from the late 1980s (Chen et al. 2009; Juillot et al. 2011). In particular, lead isotopic ratios were shown to provide a powerful tool for identifying the lead sources as this isotopic signature is specific of a given ore type and it was demonstrated to remain stable during its transfer with particles in the environment (Gulson et al. 1994; Komárek et al. 2008; Vaasjoki and Gulson 1985). In France, significant modifications of lead isotopic ratios measured in environmental samples such as airborne particles or river sediments during the last several decades were attributed to changes in anthropogenic inputs of lead (Cloquet et al. 2006a, b; Carignan et al. 2005; Ayrault et al. 2012; Monna et al. 1995). For instance, the increasing proportion of lead originating from the use of leaded gasoline was demonstrated in sediment collected during the 1990s (Elbaz-Poulichet et al. 1986; Monna et al. 1997; Véron et al. 1999). Then, after the ban of leaded gasoline in 2000, a rapid shift in isotopic signatures was found in sediment and indicated the quick and strong reduction of gasoline contribution to the total lead contamination found in the environment (Ayrault et al. 2012). Accordingly, lead isotopic ratios were measured in association with elemental geochemistry to trace potential changes in contamination sources and magnitude in sediment transiting urban catchments (Le Pape et al. 2013).

Furthermore, the transfer times of sediment and their potential spatial variations within a catchment were investigated to check whether sediment transiting the river has been recently eroded from the sources, or whether it mainly consists of material that was stored in the channel before being resuspended. To this end, sediment fingerprinting techniques based on the measurement of fallout radionuclides (FRN) characterized by different half-lives (^137^Cs, ^7^Be and ^210^Pb_xs_) have been increasingly used, although they were mainly applied in agricultural catchments (Evrard et al. 2010; Gellis et al. 2017; Le Gall et al. 2017a; Matisoff et al. 2002). These natural (^7^Be, T_1/2_ = 53 d; and ^210^Pb_xs_, T_1/2_ = 22 y) and artificial (^137^Cs, T_1/2_ = 30 y) FRN are mainly supplied to the soils by wet fallout, and they then quickly and strongly bind to fine particles (Mabit et al. 2008). The input of natural radionuclides is continuous, whereas that of radiocesium was exclusively associated with the thermonuclear bomb testing in the 1950s–1960s and the Chernobyl accident, as the fallout following Fukushima accident was shown to be negligible in France (Evrard et al. 2012). Accordingly, ^137^Cs is mainly used to discriminate between topsoil material exposed to the fallout, and subsurface material sheltered from the rainfall. In addition, the contrasting half-lives of ^7^Be and ^210^Pb are used to discriminate between material enriched in ^7^Be, reflecting their recent exposition to rainfall, and sediment depleted in ^7^Be, reflecting their longer storage in the river channel (Evrard et al. 2010, 2016; Matisoff et al. 2005). To the best of our knowledge, the current study would provide one of the first studies coupling the measurement of multiple fallout radionuclides with elemental/isotopic geochemistry in sediment transiting an urban river.

To conduct the current research, one of the most contaminated (Ayrault et al. 2014; Le Pape et al. 2012) sub-catchments of the Seine River Basin (i.e., the Orge River) was selected as it shares most characteristics of the Seine River basin (67,000 km^2^) while covering a much smaller surface area (900 km^2^). Furthermore, the Orge River catchment is exposed to a strongly increasing urban pressure in downstream direction, associated with a change in land uses (dominance of cropland and forest in upper catchment parts vs. dominance of urban areas close to the outlet) providing an ideal case study to investigate the relationship between land use and contamination processes. Finally, wastewater collected in this catchment is mainly redirected to a single treatment plant located in a nearby catchment, reducing the number of potential sources of sediment to the river (i.e., topsoil, channel banks, urban runoff).

The objectives of the current research were to define the sources and the dynamics of particles transiting the Orge River using fallout radionuclides as well as to quantify the levels and the sources of particle-bound contaminants using elemental geochemistry and lead isotopic ratios. To meet this goal, river sampling was conducted in the Orge River catchment on various occasions to cover variations of hydrological conditions. The implications of these results to improve our understanding of particle transfer in urban catchments and to guide the management of contamination sources will finally be discussed.

## Materials and methods

### Study site

**Fig. 1:**
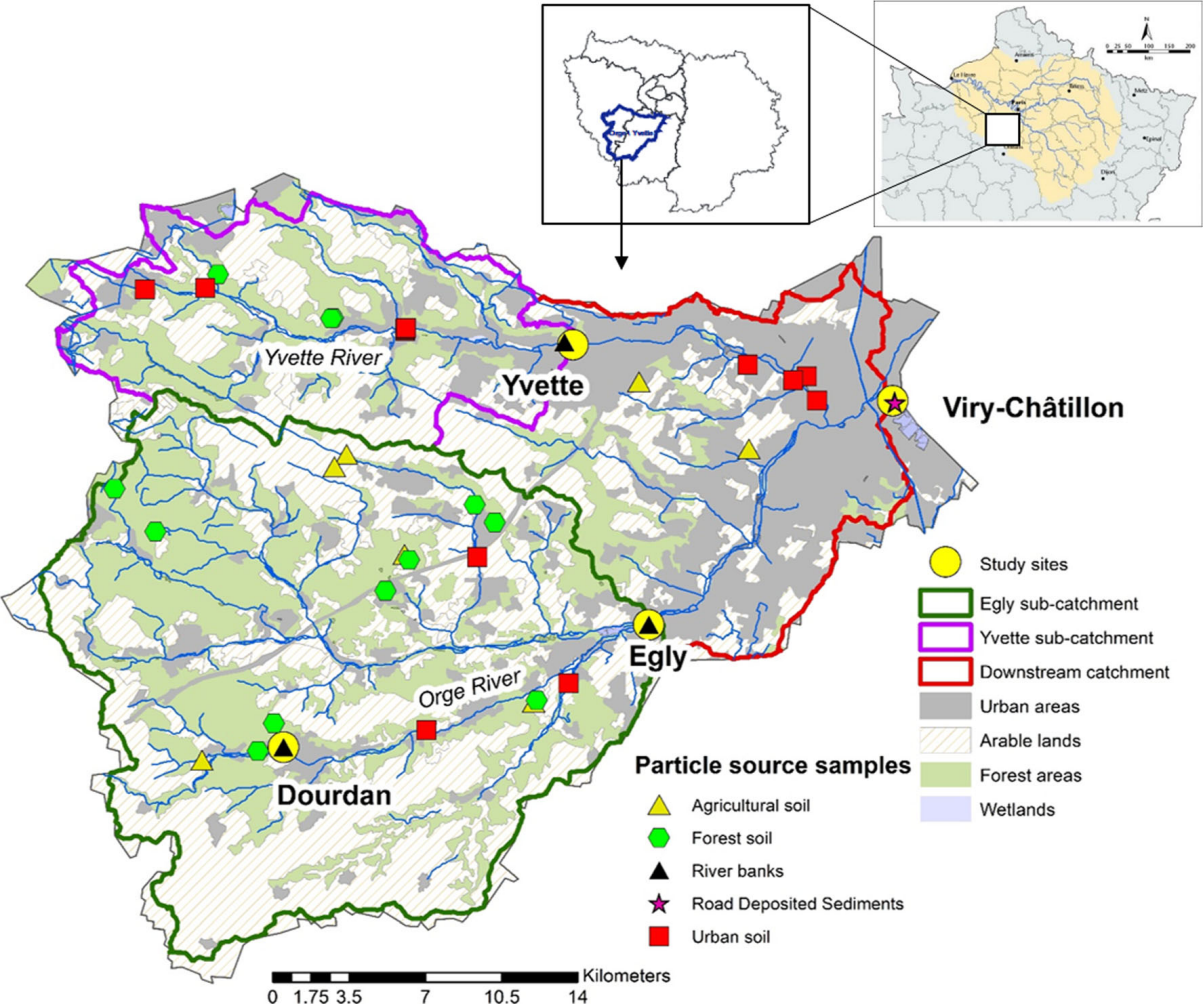
Orge River catchment: location, land use characteristics and monitoring stations (Source of the land use data: Corine Land Cover, 2012)

All samples were collected in the Orge River catchment, a sub-catchment of the Seine River Basin located 30 km south of Paris City (Fig. 1). Three sampling sites were selected on the main stem of the Orge River, whereas a fourth site was located on its main tributary, the Yvette River. The proportion of urban areas strongly increases in downstream direction, varying from 1% in upper catchment parts to 56% at the outlet (Table 1). This change is reflected by the strong increase in population densities in the drainage areas (from 300 inhabitants per km^−2^ at the most rural study site—Dourdan (“D”), to 5000 inh.km^−2^ at Viry (“V”, nearby the outlet). Although wastewater is mainly treated outside of the catchment, misconnections in the sewage network are widespread especially at the outlet, with connection failures estimated to reach 20% (SAGE Orge-Yvette 2011). Data on impervious surfaces in France for 2012 were retrieved from the European database CORINE Land Cover on land uses (Gallego et al. 2016) and used to calculate impervious surface proportions in a 2.5, 5 and 10 km radius around stations and in the sub-catchments drained by each monitoring station.

**Table 1:** Land use proportions and population densities in the Orge River sub-catchments drained by the river monitoring sites

Sub-catchments	Dourdan	Egly	Yvette	Viry (outlet)
Urban areas (%)	1	11	20	56
Agricultural land (%)	86	49	42	33
Forests (%)	12	40	38	12
Population density (inhabitants.km^−2^)	300	1400	2100	5000

Finally, the geology of the catchment is characterized by Eocene formations including carbonate rocks, marls and gypsum, and Oligocene formations dominated by Fontainebleau sands (Le Pape et al. 2012; Schneider 2005). Agricultural soils consisted of Luvisols, urban soils mainly corresponded to Fluvisols and some Luvisols, and forest soils to Planosols and Luvisols based on the FAO World Reference Base classification.

### Hydrological conditions

Sampling was conducted during a hydrological year with seven campaigns organized from June 2015 to December 2016. Water discharge at the upstream station in Dourdan (“D”) varied from 0.1 to 1.1 m^3^.s^−1^ (annual mean of 0.2 m^3^.s^−1^ from May 2015 to December 2016). At the downstream station in Viry-Châtillon (“V”), discharge ranged from 1.4 to 20.5 m^3^.s^−1^ (mean 3.3 m^3^.s^−1^). The discharge of the main tributary, i.e., the Yvette River, varied from 0.7 to 12 m^3^.s^−1^, with a mean of 1.8 m^3^ s^−1^ (May 2015–December 2016).

**Fig. 2:**
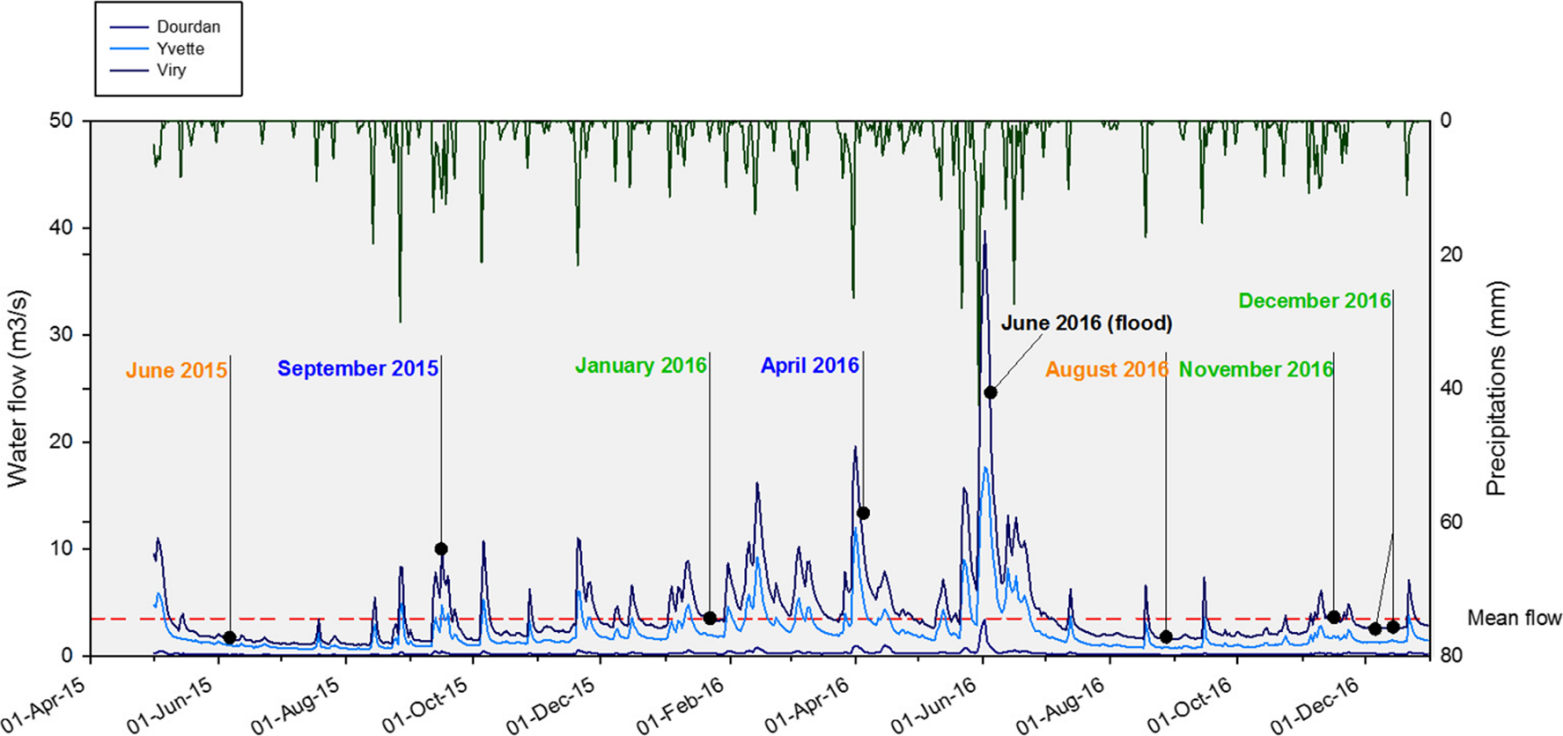
River discharge and precipitation recorded in the Orge River catchment from May 2015 to December 2016. Mean water discharge at Viry site (Red dotted line). Low flow campaigns (Orange): June 2015 (28/05/2015–03/06/2015) and August 2015 (24/08/2016–29/08/2016). Average flow campaigns (Green): January 2016 (21/01/2016–25/01/2016), November 2016 (16/11/2016–22/11/2016) and December 2016 (5/12 and 15/12/2016). High flow campaigns (Blue): September 2015 (16/09/2015–21/09/2015) and April 2016 (07/04/2016–11/04/2016). Flood campaign (Black): June 2016 (2, 3 and 6/06/2016)

Sampling campaigns were organized to cover the hydrological variations of the Orge River and their seasonality (Fig. 2). Accordingly, two campaigns were conducted during low stage periods with discharge < 2 m^3^.s^−1^ in June 2015 and August 2016 (Viry discharge records). Sampling during campaigns with discharge comprised between 3 and 4 m^3^.s^−1^ in Viry was conducted in January 2016 and November 2016. Finally, flood campaigns were organized in September 2015 and April 2016 with discharges varying between 6 and 10 m^3^.s^−1^ at the outlet.

Furthermore, sediment traps were deployed from November 22, 2016 to January 3, 2017 to compare instantaneous vs. longer term records of sediment characteristics and TE contamination.

Precipitation amounts were retrieved from national meteorological records (Météo France, www.meteofrance.com) based on daily rainfall measurements at two rain gauges, one situated in Dourdan and the second in Bretigny-sur-Orge at mid-distance between Egly and Viry-Châtillon (Fig. 1).

### Sampling and geochemical analysis

#### River sampling

Two types of samples were collected in the river: instantaneous samples of river water including dissolved and particulate phases, and integrative samples of suspended particulate matter collected during 4 to 5 days using sediment traps. The choice of sampling procedures was guided by the requirement of a significant amount of material (several grams) for conducting radionuclide measurements, difficult to achieve through the filtering of river water and justified by the representativity of sediment material collected in traps as demonstrated in several studies (Gateuille et al. 2014; Priadi et al. 2011a). Prior to the sampling, all the flasks and bottles were washed with HNO_3_ 5% and rinsed three times with ultrapure water. In the field, they were washed again several times before collecting the samples.

On the first day of each campaign, about 10 L of water was sampled at each site to collect instantaneous SPM and 1 L of additional water was sampled for analyzing the dissolved phase only. In Egly (“E”) additional samples were taken from a pipe discharging water directly into the river (E_discharge_). During each campaign, sediment traps were deployed in the river approximately 30 cm under the surface water level and submerged using ballast to collect suspended particulate matter. These traps consisted of two PET bottles of 1 L with ten drills of 2 cm diameter at their base, tied together to increase SPM collection. Four to five days later, sediment traps were collected and instantaneous river water samples were collected again. Physico-chemical parameters (pH, conductivity and temperature) were recorded during each sampling campaign.

#### Particulate source sampling

Samples representative of the potential sediment sources were collected across the catchment (Fig. 1).

First, topsoil samples were taken from A and L horizons in forests (*n* = 10), as well as in agricultural (*n* = 5) and urban (*n* = 8) areas. Most of those samples were collected in June 2015, however three samples located in Dourdan were collected in 2017. Second, the eroding face of the channel banks was sampled in Dourdan, Egly and Yvette in August 2016, at a distance of ~ 50 cm from the top, the first 2 cm were removed before sampling to avoid the collection of particles originating from topsoil erosion. Channel bank samples could not be taken in the downstream section of the river as it is channelized. Third, Road Deposited Sediments (RDS), consisting of a mixture of particles transported by urban runoff on impervious surfaces, were sampled by sweeping the road with a plastic-made brush at Viry stations in November 2016. Finally, a deep loess-derived Luvisol sample was collected at 135 cm depth in a trench dug under cropland on the Saclay Plateau, north of the Yvette River, to provide the potential background contamination signature in the study area.

#### Sample preparation

River water samples were filtered (< 0.45 μm porosity), dissolved aliquots were partly acidified (HNO_3_ 0.5 N) for subsequent TE and cation analysis and the remaining water was stored for anion, silica and carbonate analyses. Suspended particulate matter from sediment traps was centrifuged (2800 g) and freeze-dried. Suspended particulate matter and RDS were sieved to 200 μm (Pratt and Lottermoser 2007), and soil samples to 2 mm.

Approximately 100 mg of finely crushed SPM was mineralized in Teflon beakers and heated by Digiprep block (SCP Science). Three replicates were analyzed for each SPM sample collected in traps and for those instantaneous SPM samples with sufficient material available. Standard errors associated with the measurements were calculated from those analyses conducted on these three replicates.

A three-step digestion was performed with first 4 mL of HF (30%) and 2 mL HClO_4_ (67%) left at ambient temperature for 2 h then heated at 150 °C for 6 h. The second digestion phase used 3.75 mL of HCl (30%) and 1.25 mL of HNO_3_ (67%), added and kept at ambient temperature for 8 h before heating at 120 °C during 3 h20. Finally, the last part consisted of three successive evaporations for 1 h at 110 °C after addition of 1 mL of HNO_3_ (67%). The final solutions were transferred to 50 mL Falcon® (polyethylene) tubes. Each digestion included a geostandard (Lake Sediment SL1 AIEA) and a blank to control mineralization quality.

#### Geochemical analyses

##### Major elements

Major elements (Ca, Na, Mg, K, Al, Fe) were determined by Atomic Absorption Spectrometry in both mineralized SPM and dissolved phases using a VARIAN AAS240FS instrument.

##### Trace element concentrations

Minor and trace element (V, Cr, Mn, Co, Ni, Cu, Zn, As, Se, Rb, Sr, Mo, Ag, Cd, Sb, Cs, Ba, Tl) contents of mineralized particulate samples and acidified dissolved samples were measured using an Inductively Coupled Plasma Quadrupole Mass Spectrometer (ICP-QMS, X-Series, CCT II & Thermoelectron, France). A standard of river water (SRM 1640a, NIST, USA) was used to control ICP-QMS precision, and the correction of instrumental drift was based on the deviation observed on internal standards (Re, Rh and In). To avoid interferences, additional series of analyses were conducted using the Collision Cell Technology mode using gas input (H_2_ (7%) and He (93%)) in particular for elements such as Cr, Fe, Ni, Zn, As.

##### Lead isotope ratio determination

Lead isotope ratios (^206^Pb/^207^Pb and ^208^Pb/^206^Pb) were measured in solutions of mineralized samples by HR-ICP-MS (Thermo Element XR, single collector). Measurement settings were a dwell time of 10 ms, 420 sweeps, and 5 replicates per sample. A Pb reference material NIST NBS 981 was measured every three samples to control instrument drift and mass bias. The 2σ-error average of isotopic ratios was 0.14% ± 0.06 (*n* = 60). The solutions of mineralized sediment lake standard SL1 were measured with samples and presented values of 1.2177 ± 0.0035 in ^206^Pb/^207^Pb and 2.0320 ± 0.0091 in ^206^Pb/^207^Pb (*n* = 12) in agreement with those reference values of 1.217 ± 0.008 in ^206^Pb/^207^Pb and 2.037 ± 0.003 in ^208^Pb/^206^Pb (Farmer et al. 2002).

### Radionuclide measurements

Radionuclides were measured on SPM from sediment traps, RDS and channel banks samples rapidly after sampling (< 21 days). Topsoil samples collected in 2015 were analyzed in February 2017, unfortunately after the full decay of ^7^Be. In contrast, the three samples collected in May 2017 were analyzed within 2 weeks after sampling. The samples were placed in polyethylene containers and sealed airtight. The activities of ^7^Be, ^210^Pb and ^137^Cs were quantified at 447.6, 46.5 and 661.5 keV by gamma spectrometry using very low-background coaxial N and P type GeHP detectors (Ortec, Canberra) available at the Laboratoire des Sciences du Climat et de l’Environnement (LSCE) in Gif-sur-Yvette, France. The excess ^210^Pb (^210^Pb_xs_) contribution originating from atmospheric fallout was calculated by subtracting from the total ^210^Pb activity, the supported activity determined by two ^222^Ra daughters (^214^Pb and ^214^Bi) measured at 295.2 and 351.9 keV for ^214^Pb and 609.3 keV for ^214^Bi. Activities were all expressed in Bq.kg^−1^ and decay-corrected to the sampling date. Internal and certified International Atomic Energy Agency (IAEA) standard was used to verify counting efficiency and measurement reliability. The analytical errors were of ca. 10% for ^137^Cs and ^210^Pb and of 20% for ^7^Be.

### Calculation of residence time and the contribution of recently eroded particles

The residence time of particles in the river was estimated using the following Eq. (1) reported in Matisoff et al. (2005):

1 $$ t=\frac{-1}{\left({\lambda}_{7 Be}-{\lambda}_{210 Pb}\right)}\ln \left(\frac{A}{B}\right)+\frac{1}{\left({\lambda}_{7 Be}-{\lambda}_{210 Pb}\right)}\ln \left(\frac{A_0}{B_0}\right) $$

Where *λ*_7Be_ and *λ*_210Pb_ are the decay constants of respectively ^7^Be and ^210^Pb in d^−1^, A and B are ^7^Be and ^210^Pb_xs_ activities measured in SPM samples in Bq.kg^−1^ and A_0_ and B_0_ the ^7^Be and ^210^Pb_xs_ activities of recently eroded particles. In the current research, the reference value for the ratio A_0_/B_0_ was fixed to 3.3, which corresponds to that maximum ratio found in road deposited sediments collected at the Viry site.

The percentage of recently eroded particles was also calculated (Matisoff et al. 2005)(Eq. (2)):

2 $$ \% of\ recently\ eroded\ sediment=100\times \frac{\left(A/B\right)}{\left({A}_0/{B}_0\right)} $$

The reference value for A_0_/B_0_ was identical to that used in Eq. (1).

Based on ^7^Be and ^210^Pb_xs_ activities measured in SPM, a two-end-member equation was used to calculate the contribution of “older particles” with low activities in ^7^Be and ^210^Pb_xs_ (Bq.kg^−1^) and “recently tagged particles” with high activities in ^7^Be and ^210^Pb_xs_. The system of equations is detailed as follows:

3 $$ {}^7 Be SPM=x{{}^7 Be}_{old\  particles}+y{{}^7 Be}_{recent\ particles} $$

4 $$ {}^{210} Pb SPM=x{{{}^{210} Pb}_{xs}}_{old\  particles}+y{{}^{210}{Pb}_{xs}}_{recent\ particles} $$

Where *x* and *y* are the respective proportions of older particles and recently eroded particles, and their ^7^Be and ^210^Pb activities (Bq.kg^−1^). Contributions are calculated with the function *Solver* in Excel using a system of two equations with a two-step resolution.

### Statistical analysis

Statistical tests were conducted to compare geochemical and radionuclide results between defined groups of samples. First, the Shapiro-Wilk test was used to evaluate the normality of the sample distributions with a selected *α* value of 0.05 defining the *p* value limit (Shapiro and Wilk 1965). Following the result of Shapiro test, sample comparison was conducted using Student’s test (or *t* test) to compare two groups of normal distribution datasets or ANOVA to compare more than two groups. For those data with non-normal distributions, Kruskal-Wallis test was performed (Siegel and Castellan 1988). Those three tests were interpreted using an *α* value of 0.05.

## Results and discussion

### Sources of SPM and particle dynamics

#### Characterization of SPM sources

**Fig. 3:**
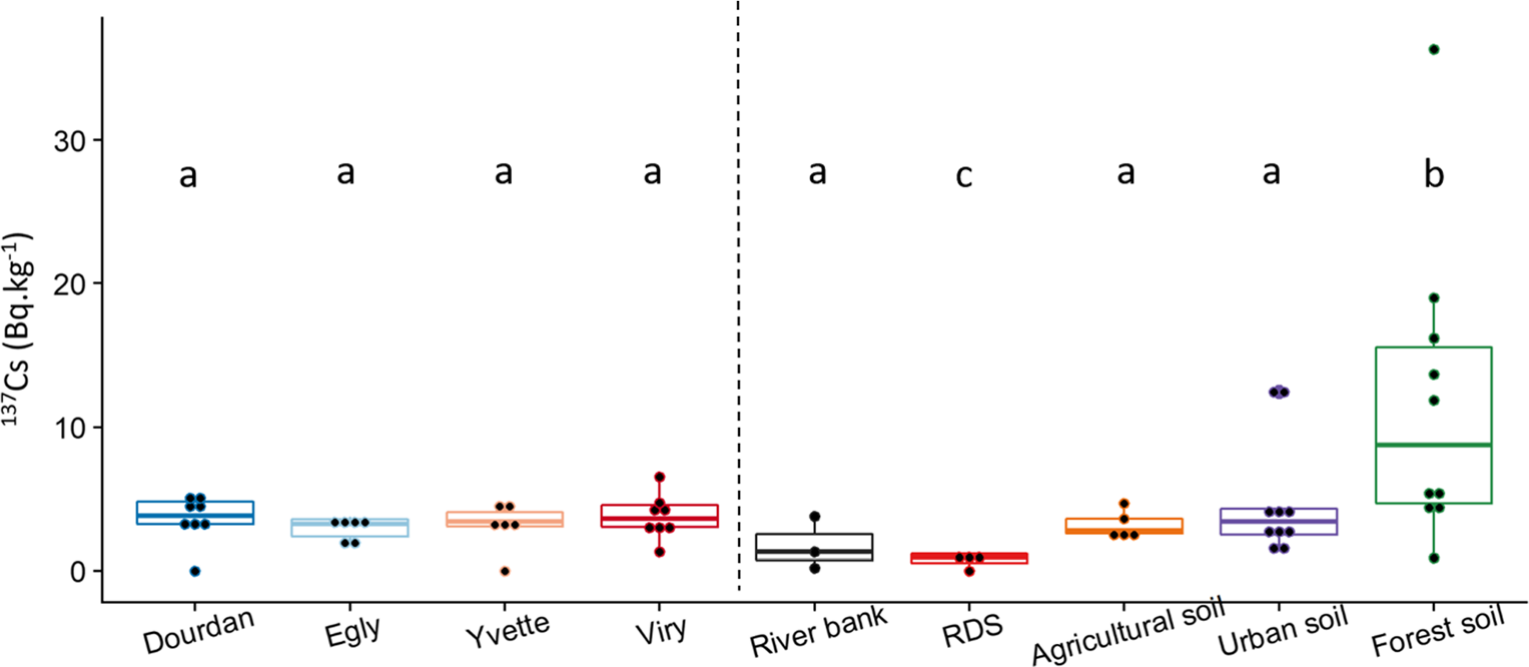
^137^Cs contents in Bq.kg^−1^ for SPM collected at D, E, Y, V, as well as in potential sources (channel bank, agricultural soil, forest soil and Road Deposited Sediment). Values characterized by the same letter (a, b or c) are not significantly different at the *α* = 0.05 level

Concentrations in ^137^Cs measured in SPM and in potential sediment sources are presented in Fig. 3, along with the results of the corresponding statistical comparison.

Suspended particulate matter from the Orge and Yvette Rivers showed relatively stable ^137^Cs activities of 3.5 ± 1.4 Bq.kg^−1^, similar to those found in most particle sources (i.e. river banks, RDS, agricultural and urban soils), with the exception of forest soils having a median activity of 8.8 Bq.kg^−1^ and a maximum value of 36.3 Bq.kg^−1^ (Table S6). Statistical tests confirmed the significance of this difference, excluding forest soils as a potential major source of particles in upper sections of the Orge River. This exclusion was corroborated by results found for major elements, with a depletion in K, Na and Mg in forest soils compared to those concentrations found in other potential sources (Fig. S1), supporting the conclusion that forest soils did not provide a major source of particles in upper catchment parts. Moreover, RDS showed low ^137^Cs activities (median 0.5 Bq.kg^−1^), suggesting that RDS did not provide a dominant source of particles. Channel banks collected along the Orge River showed lower ^137^Cs activities of 1.4 ± 1.8 Bq.kg than SPM, although they were not statistically different. Finally, agricultural and urban soils showed a ^137^Cs activity of 2.8 ± 0.8 Bq.kg^−1^ and 4.9 ± 0.2 Bq.kg^−1^ very similar to those results found in SPM. Accordingly, channel banks, agricultural soils and urban soils could not be discriminated based on their ^137^Cs activities, both providing potential significant sources of sediment to the river.

**Fig. 4:**
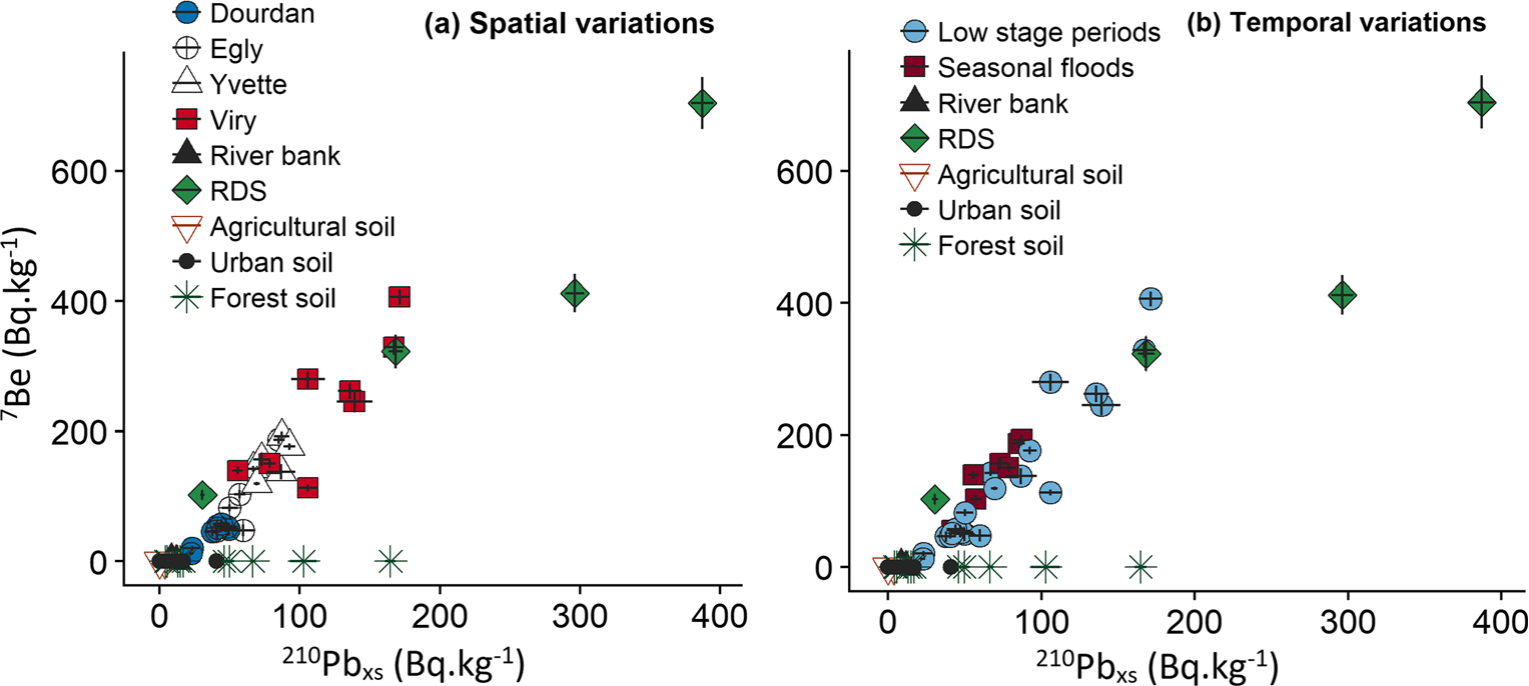
^7^Be and ^210^Pb_xs_ activities measured in SPM collected at the four river stations (**a**: Spatial variations) during contrasted hydrological regimes (**b**: Temporal variations), as well as in RDS at the outlet site Viry, and in potential sources (agricultural soils, urban soils, channel banks and forest soils)

A progressive increase in ^7^Be and ^210^Pb_xs_ concentrations was measured in SPM collected in downstream direction in the Orge River (Fig. 4a, for a plot of their spatial variations). In Dourdan, SPM signatures ranged between 12 and 57 Bq.kg^−1^ for ^7^Be and from 22 to 50 Bq.kg^−1^ for ^210^Pb_xs_. In contrast, in Viry, SPM showed much higher values varying from 113 to 406 Bq.kg^−1^ for ^7^Be and from 56 to 171 Bq.kg^−1^ for ^210^Pb_xs_. This increase in radionuclide concentrations measured in SPM indicated the increased supply of recently eroded particles in downstream direction. The suspended particulate matter signatures in ^7^Be and ^210^Pb_xs_ plot well along a mixing line between two potential particle sources (Fig. 4): the first end-ember corresponded to RDS with respective maximum values in ^7^Be and ^210^Pb_xs_ up to 704 ± 40 and 387 ± 8 Bq.kg^−1^, whereas the second end-member could not be selected between channel banks, agricultural soils and urban soils to identify the main source supplying particles to the river. However, given the virtual absence of urban soils in upper catchment parts and their low connectivity to the river network, it is very unlikely that they provided a potential major source of particles.

The strong affinity of radionuclides to the finest particle fractions including clays has been demonstrated in numerous studies (Blake et al. 2009; Davies and Shaw 1993; Matisoff et al. 2005), and corrections based on Al or Th that are enriched in the clay fraction are sometimes applied for correcting the particle size effect (Foucher et al. 2015; Sakaguchi et al. 2006). In the current research, similar Al contents were found in SPM (40 ± 8 g.kg^−1^) and agricultural soils (38 ± 11 g.kg^−1^), and they did not significantly vary from those found in RDS and river banks (24 ± 2 g.kg^−1^ and 26 ± 5 g.kg^−1^ respectively). These results demonstrate that the increasing ^7^Be and ^210^Pb_xs_ activities between soils and SPM/RDS (Fig. 4) were not related to a grain size effect, and that these activities could be used for comparing particle sources and SPM. As grain size corrections may result in a bias in source contribution calculations (Smith and Blake 2014), particle size was not corrected in the current research. During particle transfer, changes in radionuclide content could be caused by either a dilution of the signal with particles depleted in ^7^Be or by the radioactive decay during the transport. In the Orge River, the constant positive relationship between ^7^Be and ^210^Pb_xs_ in SPM suggests that the dilution effect provides the main factor explaining the difference between those sites located in upper or in lower catchment parts. If the ^7^Be significantly decayed during transport as a result of particle storage within the system, sediment would be depleted in this radioisotope resulting in SPM signatures falling outside of the mixing line actually observed (Fig. 4). The fast transfer of particles in the river system is further supported by the estimation of short SPM residence times with a mean of 63 ± 30 days (18–140 days) (Table 2), with a significant decrease observed for the particles transiting the river at Dourdan (91 ± 22 days) compared to those transported downstream at Viry (41 ± 22 days).

**Table 2:** Mean particle residence times and associated standard errors estimated for SPM samples collected at each river station

	June 2015	September 2015	January 2016	April 2016	August 2016	November 2016	December 2016	Mean value	SD
Residence time (days)									
Dourdan	105	72	94	83	140	86	78	94	23
Egly	83	32	111	48	77	55	–	68	28
Yvette	35	32	57	34	51	43	–	42	10
Viry	88	22	49	43	18	26	41	41	22

In addition to spatial variation, radionuclide content in SPM also presented variations between low stage periods (mean and low flow periods) and seasonal floods (high waters) (Fig. 4b). The changes in signatures of samples collected during seasonal floods remained limited at all stations with variations from 54 to 192 Bq.kg^−1^ in ^7^Be and 41 to 87 Bq.kg^−1^ in ^210^Pb_xs_. On the contrary, characteristics of SPM collected during low stage periods were much more scattered, with signatures varying between 12 and 406 Bq.kg^−1^ in ^7^Be and 23 to 171 Bq.kg^−1^ in ^210^Pb_xs_. During low stage periods, SPM radionuclide signatures in ^7^Be and ^210^Pb_xs_ increased strongly in downstream direction, clearly reflecting the input of recently tagged particles from urban areas in lower catchments parts. However, residence times estimated based the on ^7^Be/^210^Pb_xs_ ratios did not vary significantly between seasonal floods and low stage periods for a given site (Table 2).

#### Proportion of recently eroded particles

**Fig. 5:**
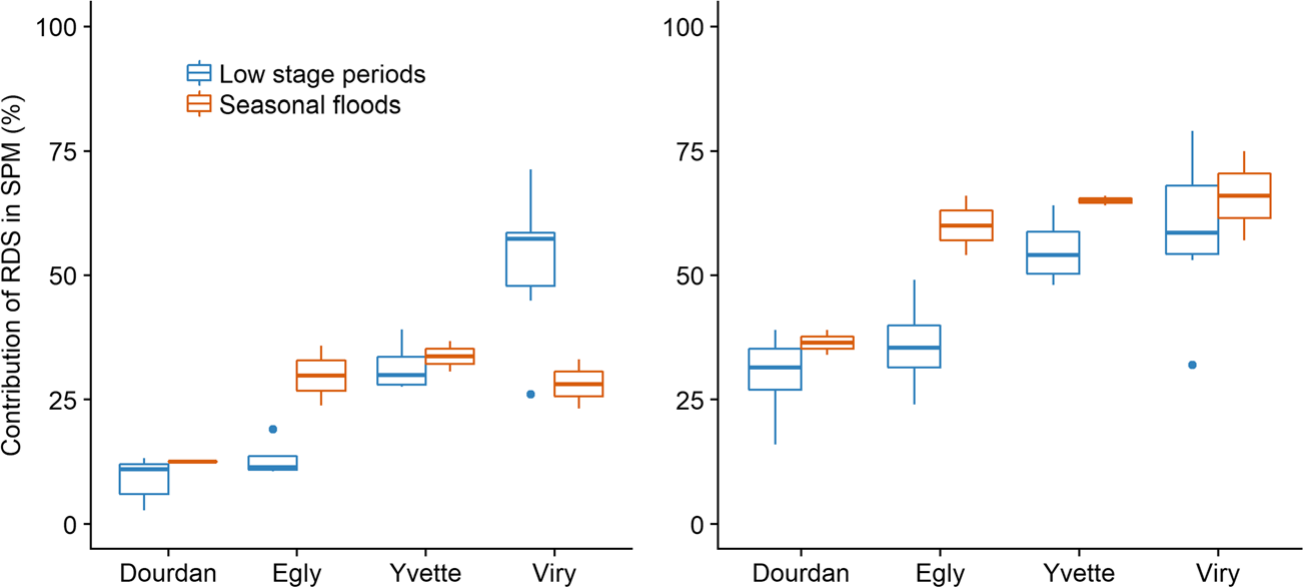
Contribution of recently eroded particles to the Orge River particles estimated with the linear mixing model (**a**) and the ^7^Be/^210^Pb_xs_ ratio model (**b**) following the increasing urbanization gradient in the catchment and for different hydrological regimes

According to the results showing a mixing line between ^7^Be and ^210^Pb_xs_ (Fig. 4), Eqs. (3) and (4) were used to estimate the contributions of particle sources. Accordingly, *older particles* were considered to originate from agricultural soils or channel banks (both showing the same signature) whereas *recent particles* were assumed to correspond to RDS. The mean ^7^Be and ^210^Pb_xs_ activities for *older particles* originating from agricultural soils (*n* = 5, Table S5) and river banks (*n* = 3, Table S5) were 1 Bq.kg^−1^ for ^7^Be and 4.0 Bq.kg^−1^ for ^210^Pb_xs_. Considering the large range of radionuclide activities found in RDS samples, the mean contributions of *recent particles* were calculated from those results obtained with the highest RDS signature (i.e., 704 Bq.kg^−1^ in ^7^Be and 387 Bq.kg^−1^ in ^210^Pb_xs_), that closest to the SPM signatures (i.e., 323 Bq.kg^−1^ in ^7^Be and 168 Bq.kg^−1^ in ^210^Pb_xs_), and the median RDS signature (i.e., 412 Bq.kg^−1^ in ^7^Be and 232 Bq.kg^−1^ in ^210^Pb_xs_) (Table S7). The mean standard error for the three sets of calculated contributions was 10%, with an increase from Dourdan (4% ± 1) to Viry (17% ± 7). The comparison of the recently eroded particle contributions estimated from the mixing line model and those calculated with the literature model based on ^7^Be/^210^Pb_xs_ ratios (see section “Statistical analysis” and Table S8) revealed a similar trend for both approaches (Fig. 5). Overall, an increasing contribution of recently eroded particles was observed in downstream direction for both models, with proportions ranging from 3 to 71% (Fig. 5a) for the mixing model and from 16 to 79% (Fig. 5b) for the ^7^Be/^210^Pb_xs_ ratio model. The main difference between both models is that the mixing model takes into account variations in the hydrological regime (Fig. 5a), which is not the case for the ^7^Be/^210^Pb_xs_ ratio model (Fig. 5b). For instance, higher contributions of recent particles were observed in Viry during low stage periods (i.e., campaigns of Jan. 2016, Nov. 2016, Dec. 2016, Jun. 2015 and Aug. 2016) with a median value of 57 ± 15%, compared to contributions during seasonal flood (i.e., Sept. 2015 and Apr. 2016) showing a median value of 28 ± 7%. Conversely, the ^7^Be/^210^Pb_xs_ ratio model did not reveal any temporal differences in the contributions of recent particles. The different pattern observed for particles collected at Egly and Yvette sites showing higher recent particle contributions during seasonal floods may reflect the current development of building activities which was shown to accelerate soil erosion and the supply of sediment to nearby rivers (Chin 2006; Huon et al. 2017; Nelson and Booth 2002). This hypothesis is supported by those lower residence times calculated for particles transiting the river at Egly and Yvette sites during seasonal floods (40 ± 11 days and 33 ± 2 days respectively) compared to those found during low stage periods (82 ± 23 days for Egly SPM and 47 ± 10 days for Yvette).

#### Impact of urban surfaces and precipitation regime on the contribution of recently tagged particles

The increasing urban pressure in downstream direction is demonstrated when comparing the proportions of urban surface areas found in a 2.5, 5 and 10 km radius from each sampling station (Table 3). Urban pressure is the highest in Viry draining the entire Orge catchment. As impervious surface rates evolution is similar for the three radius tested and comprised between 2.5 and 10 km, those values corresponding to the mid-distance of 5 km were retained for further analysis.

**Table 3:** Proportions of impervious surfaces in the area located in a 2.5, 5 and 10 km radius from each river monitoring station as well as in the drainage area of each station

Site	Impervious surfaces proportion (%)			
	2.5 km	5 km	10 km	Sub-watershed
Dourdan	8	3	2	3
Egly	19	13	11	4
Yvette	25	17	15	9
Viry	52	48	43	12

**Fig. 6:**
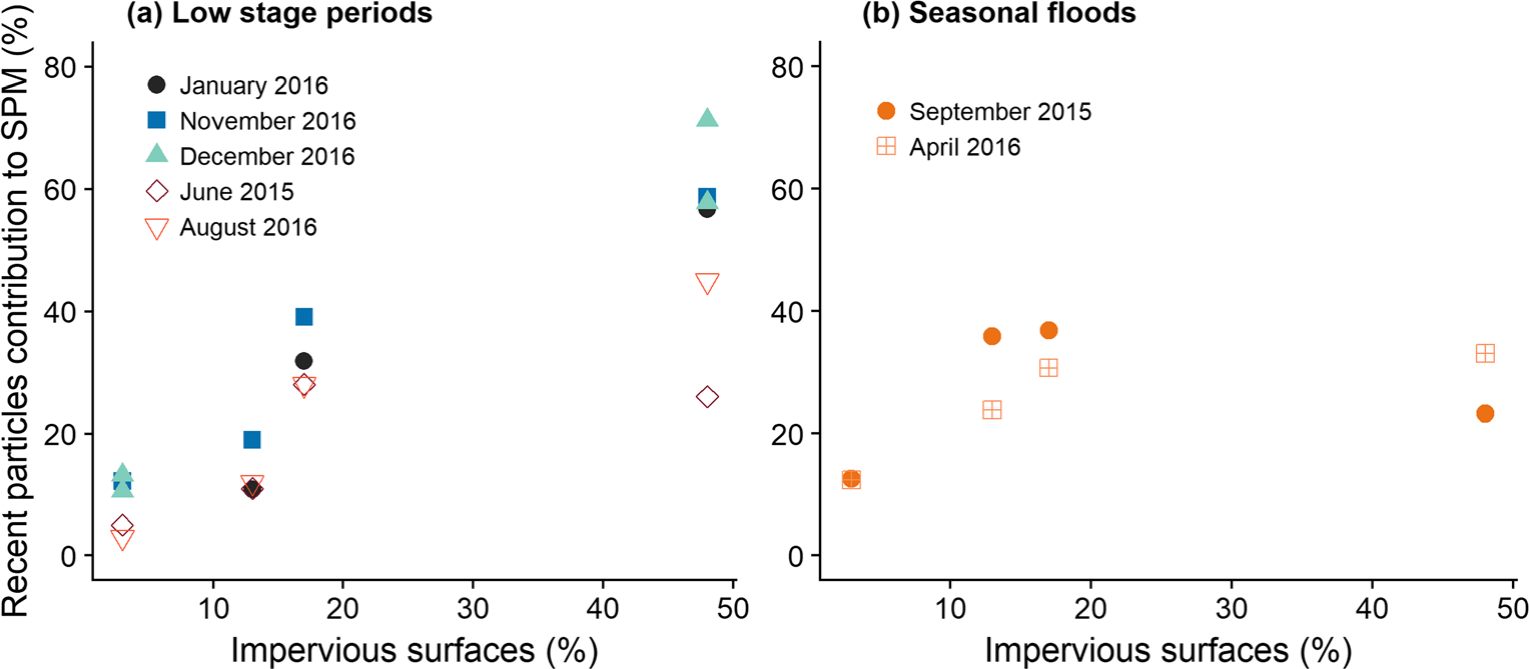
Relationship between the proportion of recently tagged particles and of impervious surfaces in a 5-km wide area around each monitoring station and for various hydrological regimes ((**a**) Low stage periods: Jan. 2016, Nov. 2016, Dec. 2016, Jun. 2015, Aug. 2016 and (**b**) Seasonal floods: Sept. 2015, Apr. 2016)

The relationship between the proportion of impervious surfaces and the contribution of recently tagged particles varied with hydrological conditions (Fig. 6). During low stage periods, a positive relationship was observed between both variables. However, contributions of recent particles showed larger variations at Viry compared to those observed at D, E and Y with an increase between low flow periods (Jun 2015, Aug 2016) and average flow periods (Jan 2016, Nov 2016 and Dec 2016). On the contrary, no relationship could be observed between the contribution of recent particles and the impervious surface areas during seasonal floods. As streamflow in the Orge River shows a rapid response to rainfall (Fig. 2), those contrasted particle transfer processes observed for different hydrological regimes (i.e., low stage periods and seasonal floods) may be explained by different timing in precipitations across the entire catchment and variations in land use patterns of each station (Table 4).

**Table 4:** Characteristics of antecedent precipitation recorded before sampling campaigns during the study period in Dourdan (upstream part) and Bretigny-sur-Orge (downstream part), with water flow measured at the outlet

	Water flow at the outlet (m^3^.s^−1^)	Antecedent precipitation during the 7 previous days (mm)		Mean precipitation per day (mm.d^−1^)		Percentage of rainy days during the 2 weeks prior to sampling (rainfall amount > 2 mm)	
		Dourdan	Bretigny-sur-Orge	Dourdan	Bretigny-sur-Orge	Dourdan	Bretigny-sur-Orge
September 2015	6.5	58.7	22.8	12	6	33	33
April 2016	6.0	24.4	33.8	5	7	25	25
June 2015	1.9	1.4	1.4	0	1	0	0
August 2016	1.7	5.2	24.5	1	8	8	17
January 2016	3.5	1.4	1.8	0	1	25	25
November 2016	3.5	5	15.3	1	3	50	50
December 2016	3.1	6	2.4	1	0	17	4

To explore this hypothesis, antecedent precipitation records were compared for the sequence of sampling campaigns.

The amount of antecedent precipitation varied depending on hydrological regimes and locations, with low stage periods occurring after very low mean precipitation records varying from 1 mm per day in upper catchments parts and up 8 mm per day in downstream parts. On the contrary, seasonal floods were triggered by higher rainfall varying between 5 and 12 mm per day (Table 4), suggesting variations in sediment dynamics depending on the hydrological regime. During seasonal floods (i.e., in Sept. 2015 and Apr. 2016), high intensity rainfall impacted differently upper and lower catchment parts. In upper parts mainly occupied by agricultural land, rainfall generated extensive soil erosion and supplied recently eroded particles to the river, particularly in Egly, resulting in low residence times of particles (Table 2, section “Proportion of recently eroded particles”). Moreover, the short and homogenous residence times found for particles at Egly, Yvette and Viry during those flood events (Table 2) reflected the occurrence of a clear connection between upstream and downstream river sections, explaining the steady contribution of recent particles observed at these three sites. During low stage periods (i.e., Jun. 2015 and Aug. 2016), soil erosion was low in upper catchment parts (Fig. 6a) as reflected by the long residence times observed in Dourdan during both campaigns and in Viry during the June 2015 campaign (Table 2). The contribution of the recently eroded particles up to 45% observed in Viry in August 2018 could be due to the occurrence of a storm that generated extensive urban runoff 1 week before the sampling (Fig. 2) whereas, in June 2015, sampling was conducted after 2 weeks of dry weather explaining the lower contribution of 26% of recent particles for this campaign. Finally, average flow periods (i.e., Jan. 2016, Nov. 2016 and Dec. 2016), characterized by frequent although low-intensity rainfall events (Fig. 2, Table 4) showed an intermediary behavior. In upper parts, the low contribution of recent particles (Fig. 6) reflected low soil erosion, supported by a longer residence time of particles (Table 2), whereas the highest contribution of recently eroded particles (Fig. 6) was found in lower river sections at Viry, reflecting the higher contribution of urban runoff in this area.

### Contamination sources and magnitude

#### Local background

The local background signature was determined by the current research for the Orge catchment (Table 5). The comparison with the literature shows the similarity of these values with those estimated for the entire Seine Basin background. The latter values were estimated based on the analysis of riverbed sediment samples collected in 56 small rural, agricultural and forested catchments across the Seine River basin and two additional riverbed sediment samples collected in an archeological site (aged of 5000 BP) in Bercy (Paris *intramuros*) (Horowitz et al. 1999; Thévenot et al. 2002). These background values were used in numerous studies investigating the metallic contamination of sediment transiting the Seine River (Le Cloarec et al. 2011; Meybeck et al. 2007; Thévenot et al. 2007).

**Table 5:** Comparison of the metal background values defined for the Seine River (Thévenot et al. 2002) for the Orge River (this study) and the Upper Continental Crust (Taylor and McLennan, 1995)

	Cr	Co	Ni	Cu	Zn	Sb	Pb	Al
mg.kg^−1^								
Luvisol (Saclay plateau soil)	69	10	31	11	74	0.6	18	49,700
Seine background^a,b^	40	9	16	15	60	0.8	20	33,000
Upper Continental Crust^c^	35	10	20	25	71	0.2	20	80,400

^a^Thévenot et al. 2002

^b^Le Cloarec et al. 2011

^c^Taylor & Mclennan 1995

The values of 11, 74, 0.6, 18 and 10 mg.kg^−1^ respectively in Cu, Zn, Sb, Pb and Co measured in the Luvisol of the Orge River catchment are similar with both UCC values and the Seine River background. However, higher values in Cr and Ni of 69 and 32 mg.kg^−1^, found in the Orge River catchment, could indicate a contamination of the Luvisol by agricultural fertilizers especially for Cr (Le Pape et al. 2012).

#### Contamination of suspended particulate matter and particle sources

Enrichment factors (EF) were calculated (Chester and Stoner 1973) based on trace element concentrations in SPM, agricultural soils, river bank and RDS provided in the Supplementary Material (Tables S1 and S3 for SPM, Table S5 for potential particle sources):

5 $$ EF=\frac{{\left[ TE\right]}_{SPM}/{\left[ Al\right]}_{SPM}}{{\left[ TE\right]}_{background}/{\left[ Al\right]}_{background}} $$

With [TE] the trace element concentration and [Al] the aluminum concentration in in mg.kg^−1^ measured in SPM and in the local Orge geochemical background as defined in Table 5.

Trace element concentrations and EF calculated in SPM collected in the Orge River (Table S1 and 3) did not show any significant temporal variation throughout seasons or for different hydrological regimes.

In contrast, spatial variations were significant and they showed that changes in EF in SPM followed two trends. First, Cu, Zn, Sb, Pb showed increasing EF values in downstream direction, with values ranging from 3 to 9 for Cu, 2 to 7 for Zn, 3 to 6 for Pb and 2 to 7 for Sb. Significance of the differences observed between sites was verified with a Kruskal-Wallis test and conclusive for all four contaminants. According to the literature (Sutherland 2000; Szuszkiewicz et al. 2016), an EF lower than 2 indicates a minimal contamination, moderate contamination corresponds to EF varying between 2 and 5 and significant contamination is observed when EF exceed 5. Accordingly, SPM from the Orge River catchment appears to be moderately contaminated in Dourdan, Egly, Yvette, whereas it is considered to be significantly contaminated in Viry for Cu, Zn, Sb and Pb. Furthermore, a significant correlation was observed between Cu-Zn-Sb-Pb with *r*^2^ > 0.9 (Table S10) suggesting the supply of these four contaminants by a common source likely located in urban areas found in the lower catchment parts. Second, Ni, Co and Cr showed stable EF values ~ 1 throughout time for all three contaminants, which likely indicates that these elements are provided by the geochemical background, and no enrichment is observed in downstream direction.

**Fig. 7:**
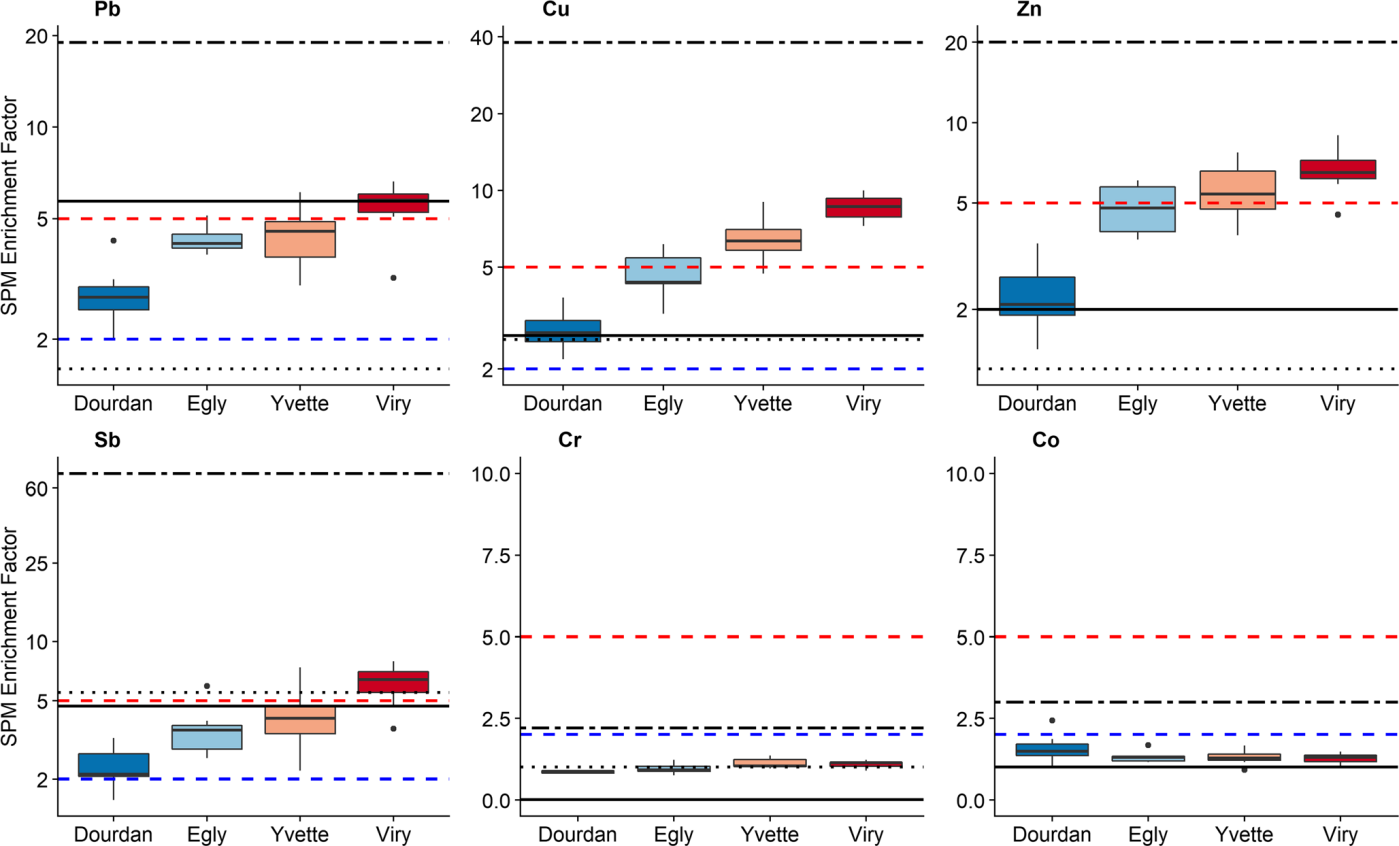
Enrichment factors in trace elements in suspended particulate matter collected at the 4 monitoring stations in the Orge River along with EF of agricultural soils (black dotted line), river bank samples (black solid line), and RDS (black double dashed line). Blue dashed line: lower limit of moderate contamination; Red dashed line: lower limit of the significant contamination level

Agricultural soils showed EF in Pb, Cu, Zn of respectively 2,3 and 1, which were lower than those found in particles transiting the Orge River at the most upstream site in Dourdan (Fig. 7). In contrast, a significant contamination in Sb was observed in agricultural soils, with an EF exceeding 5. As a comparison, channel banks showed a significant contamination in Pb with an EF of 6 (Table S5), and EF similar to those found in agricultural soils for Cu, Zn and Sb. Finally, road deposited sediments presented very high EF, beyond the range of those found in the Orge SPM (Fig. 7), of 19 ± 7 for Pb, 38 ± 13 for Cu, 20 ± 11 for Zn and 71 ± 34 for Sb (Table S5). Despite those disparities, agricultural soils and river banks showed EF in Co and Cr similar to those found in SPM values, whereas slightly higher values were observed for RDS samples being moderately contaminated. Accordingly, contamination rates found in RDS were in agreement with those results based on radionuclide concentrations and demonstrated that urban areas provide a significant source of contaminated particles.

#### Lead isotopic signature of SPM and potential sources

**Fig. 8:**
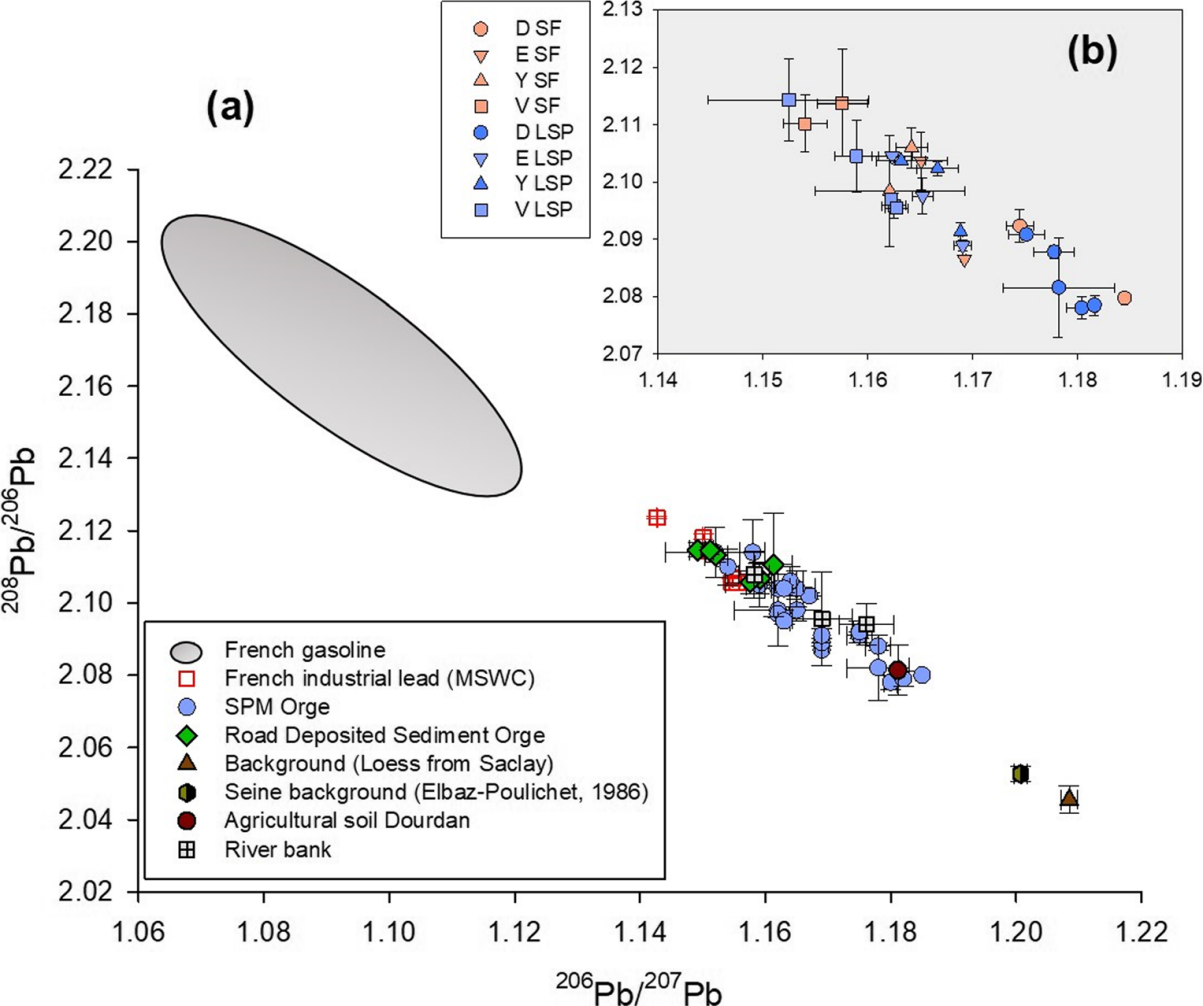
Lead isotopic signatures found in contrasted soil/sediment samples collected in the Orge River catchment and in potential sources: (**a**) Lead isotopic signatures of French gasoline (Monna et al. 1997; Roy 1996); Industrial lead estimated from French Municipal Solid Waste Combustors from Sete and Paris (Monna et al. 1997; Widory et al. 2004); Road Deposit from the Orge (this study); Suspended Particulate Matter from the Orge River (this study); Local background (this study); Agricultural soil from Dourdan (this study) and channel bank (this study). (**b**) SPM lead signatures obtained at each river monitoring site (Dourdan (D): circle, Egly (E): inverted triangle, Yvette (Y): triangle, Viry (V): square) and for contrasted hydrological regimes (seasonal floods (SF): orange, low stage periods (LSP): blue)

All the lead isotope ratios are reported in Fig. 8 and in Supplementary Material (Tables S2 and S10).

Lead isotopic ratios measured in SPM varied between 1.1845 ± 0.0010 and 1.1525 ± 0.0077 for ^206^Pb/^207^Pb, and from 2.0780 ± 0.0019 to 2.1143 ± 0.0071 for ^208^Pb/^206^Pb. Lead isotopic signatures in SPM plotted along a single line in the three isotope space diagram (Fig. 8), which indicates that the SPM signature results from a simple binary mixing of two sources of lead: a “natural source” and an “urban source.” After the quick decrease of the gasoline contribution to the lead signature since 2000 (Ayrault et al. 2012; Le Pape et al. 2013), the results of the current research confirm that leaded gasoline is not a dominant contamination source any more in the sediment transiting the Orge River more than 15 years after the ban.

The local background measured in the current research (Table 4) provides relevant values to calculate the natural background contribution. These background values remain close to those estimated for the Seine River background (Elbaz-Poulichet et al. 1986). In contrast, RDS showed isotopic signatures similar to those found in municipal solid waste combustors (MSWC) samples collected in Paris and Sète (southern France). The characteristics of MSWC fly ashes are considered to provide an “urban lead signature” (Ayrault et al. 2012; Carignan et al. 2005; Cloquet et al. 2006a, 2006b; Monna et al. 1997). The similar values found for French MSWC and RDS collected in the Orge River catchment confirm the relevance of using the latter values to characterize “urban lead” contaminating SPM transiting the Orge River.

These values suggest that RDS provide the major source of lead contamination to SPM transiting downstream sections of the Orge River. This result corroborates the findings derived from radionuclide measurements (section “Characterization of SPM sources”). Finally, the lead isotopic compositions provide a tool to discriminate between agricultural soil and channel banks that were characterized by similar radionuclide activities. Agricultural soils were characterized by higher ^206^Pb/^207^Pb signatures (1.1811 ± 0.0003) compared to channel banks (1.1761 ± 0.0043 to 1.1582 ± 0.0024). Furthermore, lower ^208^Pb/^206^Pb ratios were found in an agricultural soil sample (2.0813 ± 0.0069) compared to channel banks (2.0940 ± 0.0057 to 2.1079 ± 0.0065). The signatures measured in the SPM collected at the upstream site (Dourdan) were very close to that of agricultural soil (Fig. 8a), suggesting that cropland provided the major source of particles supplied to upper sections of the Orge River.

Finally, lead isotopic compositions in SPM did not change significantly for various hydrological regimes (low stage waters vs. seasonal floods), demonstrating the occurrence of a steady source of Pb contamination in the Orge River catchment despite those variations in particle transfer dynamics illustrated by changes in radionuclide signatures.

## Conclusions

The current research demonstrated based on multiple fallout radionuclide measurements that road deposited sediments (RDS) provided a significant source of particles transiting an urban river representative of those tributaries found in the Seine River basin, France, and in similar regions of the world characterized by extensive legacy contamination. RDS were also shown to supply a major source of particle-bound contamination to the river based on the examination of lead isotopic signatures. Furthermore, fallout radionuclide signatures indicated that the contribution of sediment supplied by urban runoff increased when larger urban surfaces were drained, although variations were observed for contrasting hydrological regimes. This trend was mainly observed during low stage periods with a dominant contribution of urban areas, whereas a homogenization in SPM signatures was found during seasonal floods with higher contributions of agricultural soils and channel banks. This research also demonstrated the interest of coupling measurements of fallout radionuclides, elemental and isotopic geochemistry to trace both particle-bound contamination sources and sediment dynamics in urban catchments. In the future, this type of sediment tracing techniques could usefully be applied to organic pollutants such as polycyclic aromatic hydrocarbons (PAH). This would improve our understanding of their complex transfer in the environment and guide the implementation of effective management measures in order to reduce the deleterious impacts generated by the supply of both organic and inorganic contaminants to urban rivers.

## Electronic supplementary material

ESM 1 (DOCX 385 kb)
