# Supplementary material for: Tracing the sources of suspended sediment and particle-bound trace metal elements in an urban catchment coupling elemental and isotopic geochemistry, and fallout radionuclides
Source: Environ Sci Pollut Res Int. 2018 Aug 10;25(28):28667–81. doi: 10.1007/s11356-018-2892-3 (PMC6153679; doi:10.1007/s11356-018-2892-3)
Supplement: Supplementary file 1 — (DOCX 385 kb) [file 11356_2018_2892_MOESM1_ESM.docx]

Supplementary material

Table S1. Aluminium and trace element concentrations measured in SPM collected in the Orge River between June 2015 and December 2016. D: Dourdan, E: Egly, Y: Yvette, V: Viry

| Sample | Campaign | Season | Al | Cr | | Co | | Ni | | Cu | | Zn | | Ag | | Sb | | Pb | |
| --- | --- | --- | --- | --- | --- | --- | --- | --- | --- | --- | --- | --- | --- | --- | --- | --- | --- | --- | --- |
|  |  |  | **g.kg^-1^** | **mg.kg^-1^** | | | | | | | | | | | | | | | |
|  | **Limits of detection** |  | **0.1** | **2.4** | | **0.01** | | **0.3** | | **0.2** | | **1.1** | | **0.002** | | **0.1** | | **0.01** | |
|  |  |  |  | **mean** | **SD** | **mean** | **SD** | **mean** | **SD** | **mean** | **SD** | **mean** | **SD** | **mean** | **SD** | **mean** | **SD** | **mean** | **SD** |
| D1 | June 2015 | Summer | 50.4 | 64.0 | - | 15.0 | - | 35.0 | - | 32.0 | - | 141.0 | - | 0.2 | - | 1.3 | - | 42.0 | - |
| D2 | September 2015 | Summer | 40.1 | 47.3 | 0.0 | 8.1 | 0.3 | 20.7 | 0.2 | 20.1 | 0.4 | 84.8 | 9.7 | 0.2 | 0.01 | 1.0 | 0.10 | 29.0 | 0.1 |
| D3 | January 2016 | Winter | 44.8 | 49.4 | 0.3 | 12.8 | 0.2 | 27.8 | 0.3 | 25.7 | 0.2 | 128.8 | 0.3 | 0.3 | 0.02 | 1.3 | 0.05 | 44.2 | 1.5 |
| D4 | April 2016 | Summer | 39.0 | 48.0 | 1.1 | 13.9 | 0.1 | 30.3 | 0.2 | 25.8 | 0.2 | 125.3 | 2.4 | 0.1 | 0.00 | 1.5 | 0.02 | 44.6 | 0.8 |
| D5 | August 2016 | Summer | 50.9 | 58.2 | 0.4 | 13.6 | 0.2 | 34.4 | 0.3 | 30.4 | 0.6 | 158.4 | 1.0 | 0.2 | 0.04 | 1.3 | 0.04 | 50.7 | 0.4 |
| D6 | November 2016 | Winter | 34.6 | 44.0 | 0.9 | 16.1 | 0.4 | 28.0 | 0.4 | 26.2 | 0.2 | 181.2 | 2.1 | 0.2 | 0.01 | 1.3 | 0.01 | 53.0 | 2.2 |
| D7 | December 2016 | Winter | 41.7 | 44.8 | 0.6 | 12.2 | 0.1 | 27.0 | 0.2 | 24.7 | 1.0 | 137.5 | 1.6 | 0.3 | 0.13 | 1.0 | 0.03 | 46.3 | 0.8 |
| D8 | December 2016 | Winter | 43.5 | 47.5 | 0.1 | 12.7 | 0.1 | 28.4 | 0.9 | 24.8 | 0.5 | 136.2 | 0.7 | 0.2 | 0.01 | 1.0 | 0.08 | 45.8 | 0.5 |
| E1 | June 2015 | Summer | 53.7 | 91.0 |  | 14.0 |  | 40.0 |  | 76.0 |  | 448.0 |  | 0.6 |  | 2.6 |  | 74.0 |  |
| E2 | September 2015 | Summer | 35.4 | 49.2 | 2.5 | 8.9 | 0.3 | 24.1 | 0.8 | 43.8 | 0.5 | 251.9 | 9.5 | 0.4 | 0.00 | 2.5 | 0.16 | 55.9 | 1.7 |
| E3 | January 2016 | Winter | 40.3 | 47.8 | 0.5 | 9.1 | 0.2 | 21.9 | 0.4 | 40.2 | 1.4 | 229.9 | 6.0 | 0.4 | 0.05 | 1.4 | 0.07 | 56.8 | 1.6 |
| E4 | April 2016 | Summer | 37.0 | 47.0 | 0.2 | 9.2 | 0.1 | 22.2 | 0.2 | 36.3 | 1.1 | 201.8 | 0.9 | 0.3 | 0.01 | 1.6 | 0.09 | 55.2 | 0.7 |
| E5 | August 2016 | Summer | 43.2 | 54.4 | 0.4 | 9.9 | 0.1 | 25.3 | 0.1 | 43.4 | 0.8 | 255.9 | 0.9 | 0.5 | 0.03 | 1.5 | 0.02 | 64.8 | 0.2 |
| E6 | November 2016 | Winter | 29.9 | 43.4 | 0.3 | 9.6 | 0.2 | 20.0 | 0.1 | 37.8 | 0.7 | 262.3 | 7.7 | 0.3 | 0.02 | 1.3 | 0.05 | 55.5 | 0.9 |
| Y1 | June 2015 | Summer | 39.6 | 53.0 |  | 11.0 |  | 28.0 |  | 43.0 |  | 252.0 |  | 0.4 |  | 1.4 |  | 51.0 |  |
| Y2 | September 2015 | Summer | 20.7 | 38.8 | 0.4 | 6.6 | 0.2 | 20.3 | 0.2 | 42.8 | 1.1 | 238.3 | 5.9 | 0.4 | 0.00 | 1.9 | 0.33 | 45.7 | 1.1 |
| Y3 | January 2016 | Winter | 44.3 | 62.6 | 0.4 | 9.8 | 0.0 | 26.2 | 0.0 | 60.5 | 0.8 | 347.1 | 2.0 | 0.6 | 0.01 | 2.1 | 0.04 | 62.7 | 0.7 |
| Y4 | April 2016 | Summer | 34.8 | 50.3 | 0.5 | 8.4 | 0.0 | 22.8 | 0.7 | 52.3 | 0.7 | 279.4 | 0.4 | 0.4 | 0.01 | 2.0 | 0.07 | 57.2 | 0.5 |
| Y5 | August 2016 | Summer | 41.0 | 73.4 | 0.6 | 10.0 | 0.0 | 29.2 | 0.3 | 71.0 | 0.5 | 385.4 | 4.5 | 0.7 | 0.10 | 2.2 | 0.08 | 75.5 | 0.8 |
| Y6 | November 2016 | Winter | 38.6 | 63.5 | 3.7 | 9.9 | 0.3 | 26.2 | 0.7 | 56.3 | 1.4 | 393.5 | 14.6 | 0.5 | 0.07 | 1.9 | 0.10 | 65.9 | 1.0 |
| V1 | June 2015 | Summer | 42.4 | 72.0 |  | 12.0 |  | 33.0 |  | 84.0 |  | 408.0 |  | 1.9 |  | 2.4 |  | 78.0 |  |
| V2 | September 2015 | Summer | 21.0 | 28.3 | 4.0 | 4.5 | 0.9 | 12.1 | 1.4 | 38.7 | 8.2 | 202.8 | 36.2 | 0.5 | 0.07 | 1.9 | 0.31 | 43.7 | 5.9 |
| V3 | January 2016 | Winter | 49.6 | 74.5 | 3.8 | 11.6 | 0.1 | 30.3 | 0.3 | 112.8 | 1.2 | 554.0 | 3.0 | 3.3 | 0.04 | 3.8 | 0.04 | 97.1 | 1.0 |
| V4 | April 2016 | Summer | 35.4 | 55.4 | 2.8 | 8.8 | 0.1 | 21.6 | 0.3 | 62.9 | 0.8 | 310.2 | 12.9 | 1.0 | 0.07 | 2.7 | 0.07 | 75.0 | 5.9 |
| V5 | August 2016 | Summer | 37.0 | 59.6 | 2.2 | 9.4 | 0.1 | 24.0 | 0.6 | 74.3 | 2.4 | 381.6 | 2.7 | 1.5 | 0.21 | 3.0 | 0.07 | 88.6 | 10.6 |
| V6 | November 2016 | Winter | 37.0 | 58.6 | 1.4 | 10.0 | 0.2 | 23.1 | 0.4 | 84.9 | 1.1 | 493.8 | 1.6 | 1.5 | 0.15 | 3.6 | 0.12 | 83.2 | 1.2 |
| V7 | December 2016 | Winter | 39.6 | 56.4 | 0.3 | 8.9 | 0.3 | 22.3 | 0.5 | 83.2 | 1.8 | 435.0 | 19.6 | 1.3 | 0.04 | 3.3 | 0.09 | 83.6 | 3.8 |
| V8 | December 2016 | Winter | 45.0 | 63.1 | 0.8 | 9.8 | 0.0 | 25.5 | 0.2 | 87.2 | 0.2 | 439.0 | 0.2 | 1.5 | 0.10 | 3.2 | 0.17 | 89.3 | 5.0 |

| Sample | Campaign | Season | ^206^Pb/^207^Pb | | ^208^Pb/^206^Pb | |
| --- | --- | --- | --- | --- | --- | --- |
|  |  |  |  | |  | |
|  |  |  |  | **SD** |  | **SD** |
| D1 | June 2015 | Summer | n.d. | n.d. | n.d. | n.d. |
| D2 | September 2015 | Summer | **1.1845** | n.d. | **2.0797** | n.d. |
| D3 | January 2016 | Winter | 1.1752 | 0.0017 | 2.0909 | 0.0009 |
| D4 | April 2016 | Summer | 1.1745 | 0.0013 | 2.0923 | 0.0029 |
| D5 | August 2016 | Summer | 1.1778 | 0.0019 | 2.0878 | 0.0011 |
| D6 | November 2016 | Winter | 1.1782 | 0.0053 | 2.0816 | 0.0086 |
| D7 | December 2016 | Winter | 1.1805 | 0.0015 | 2.0780 | 0.0019 |
| D8 | December 2016 | Winter | 1.1817 | 0.0005 | 2.0785 | 0.0017 |
| E1 | June 2015 | Summer | n.d. | n.d. | n.d. | n.d. |
| E2 | September 2015 | Summer | **1.1693** | n.d. | **2.0865** | n.d. |
| E3 | January 2016 | Winter | 1.1653 | 0.0010 | 2.0975 | 0.0031 |
| E4 | April 2016 | Summer | 1.1651 | 0.0025 | 2.1037 | 0.0050 |
| E5 | August 2016 | Summer | 1.1624 | 0.0020 | 2.1045 | 0.0004 |
| E6 | November 2016 | Winter | 1.1691 | 0.0009 | 2.0889 | 0.0010 |
| Y1 | June 2015 | Summer | n.d. | n.d. | n.d. | n.d. |
| Y2 | September 2015 | Summer | 1.1621 | 0.0072 | 2.0984 | 0.0097 |
| Y3 | January 2016 | Winter | 1.1667 | 0.0020 | 2.1023 | 0.0013 |
| Y4 | April 2016 | Summer | 1.1642 | 0.0015 | 2.1060 | 0.0035 |
| Y5 | August 2016 | Summer | 1.1632 | 0.0024 | 2.1037 | 0.0006 |
| Y6 | November 2016 | Winter | 1.1689 | 0.0002 | 2.0913 | 0.0015 |
| V1 | June 2015 | Summer | n.d. | n.d. | n.d. | n.d. |
| V2 | September 2015 | Summer | 1.1541 | 0.0021 | 2.1102 | 0.0049 |
| V3 | January 2016 | Winter | 1.1590 | 0.0021 | 2.1045 | 0.0063 |
| V4 | April 2016 | Summer | 1.1576 | 0.0024 | 2.1137 | 0.0093 |
| V5 | August 2016 | Summer | 1.1525 | 0.0077 | 2.1143 | 0.0071 |
| V6 | November 2016 | Winter | 1.1625 | 0.0012 | 2.0959 | 0.0022 |
| V7 | December 2016 | Winter | 1.1623 | 0.0003 | 2.0969 | 0.0005 |
| V8 | December 2016 | Winter | 1.1628 | 0.0011 | 2.0955 | 0.0009 |

Table S2. Lead isotope ratios and associated standard deviation (SD, 2 σ) measured in the Orge River SPM

**Ratios in bold:** insufficient material available to analyze replicates; n.d. not determined

Table S3. SPM enrichment factors (EF) in Cu, Zn, Pb and Sb. D: Dourdan, E: Egly, Y: Yvette, V: Viry

| Sample | Campaign | EF Cu | EF Zn | EF Pb | EF Sb |
| --- | --- | --- | --- | --- | --- |
| D1 | June 2015 | 3 | 2 | 2 | 2 |
| D2 | September 2015 | 2 | 1 | 2 | 2 |
| D3 | January 2016 | 2 | 2 | 3 | 2 |
| D4 | April 2016 | 3 | 2 | 3 | 3 |
| D5 | August 2016 | 3 | 2 | 3 | 2 |
| D6 | November 2016 | 3 | 4 | 4 | 3 |
| D7 | December 2016 | 3 | 2 | 3 | 2 |
| D8 | December 2016 | 2 | 2 | 3 | 2 |
| E1 | June 2015 | 6 | 6 | 4 | 4 |
| E2 | September 2015 | 5 | 5 | 4 | 6 |
| E3 | January 2016 | 4 | 4 | 4 | 3 |
| E4 | April 2016 | 4 | 4 | 4 | 4 |
| E5 | August 2016 | 4 | 4 | 4 | 3 |
| E6 | November 2016 | 5 | 6 | 5 | 4 |
| Y1 | June 2015 | 5 | 4 | 4 | 3 |
| Y2 | September 2015 | 9 | 8 | 6 | 7 |
| Y3 | January 2016 | 6 | 5 | 4 | 4 |
| Y4 | April 2016 | 6 | 5 | 5 | 5 |
| Y5 | August 2016 | 7 | 6 | 5 | 4 |
| Y6 | November 2016 | 6 | 7 | 5 | 4 |
| V1 | June 2015 | 8 | 6 | 5 | 5 |
| V2 | September 2015 | 8 | 6 | 6 | 7 |
| V3 | January 2016 | 10 | 8 | 5 | 6 |
| V4 | April 2016 | 8 | 6 | 6 | 6 |
| V5 | August 2016 | 9 | 7 | 7 | 7 |
| V6 | November 2016 | 10 | 9 | 6 | 8 |
| V7 | December 2016 | 9 | 7 | 6 | 7 |
| V8 | December 2016 | 8 | 7 | 5 | 6 |

Table S4. Radionuclide activities in suspended particulate matter (SPM) collected in the Orge River. D: Dourdan E: Egly, Y: Yvette, V: Viry

| Sample | Campaign | ^137^Cs | SD | ^210^Pb_xs_ | SD | ^7^Be | SD |
| --- | --- | --- | --- | --- | --- | --- | --- |
|  |  | Bq.kg^-1^ | | | | | |
|  | **Limits of detection** | 0.3 – 0.5 | | 3.4 ± 1.2 | | 3 ± 0.7 | |
| D1 | June 2015 | 4.2 | 0.5 | 23.3 | 5.8 | 19.9 | 4.3 |
| D2 | September 2015 | 4.8 | 0.3 | 41.7 | 3.1 | 54.9 | 4.2 |
| D3 | January 2016 | <DL | - | 49.7 | 6.9 | 49.5 | 10.1 |
| D4 | April 2016 | 3.0 | 0.8 | 47.3 | 7.0 | 54.1 | 9.1 |
| D5 | August 2016 | 3.3 | 0.2 | 22.7 | 1.7 | 12.4 | 1.3 |
| D6 | November 2016 | 5.1 | 0.8 | 48.3 | 7.4 | 52.8 | 6.6 |
| D7 | December 2016 | 5.0 | 0.7 | 37.8 | 5.8 | 46.0 | 7.7 |
| D8 | December 2016 | 3.5 | 0.7 | 44.2 | 6.0 | 57.3 | 7.2 |
| E1 | June 2015 | 3.5 | 0.3 | 40.6 | 3.9 | 46.2 | 3.2 |
| E2 | September 2015 | 3.7 | 0.4 | 85.3 | 4.1 | 187.0 | 6.9 |
| E3 | January 2016 | 1.7 | 0.7 | 59.9 | 7.4 | 47.6 | 9.3 |
| E4 | April 2016 | 2.2 | 0.4 | 57.4 | 4.1 | 103.2 | 6 |
| E5 | August 2016 | 3.0 | 0.2 | 41.6 | 2.1 | 51.3 | 2.1 |
| E6 | November 2016 | 3.6 | 0.6 | 50.2 | 5.6 | 82.2 | 5.6 |
| Y1 | June 2015 | 4.3 | 0.3 | 67.0 | 3.8 | 142.4 | 4.2 |
| Y2 | September 2016 | 3.4 | 0.5 | 87.2 | 5.3 | 192.4 | 7.7 |
| Y3 | January 2016 | 4.7 | 1.1 | 86.7 | 10.4 | 137.7 | 12.2 |
| Y4 | April 2016 | <DL | - | 73.0 | 6.1 | 156.9 | 10 |
| Y5 | August 2016 | 3.0 | 0.2 | 69.5 | 2.1 | 119.3 | 2.6 |
| Y6 | November 2016 | 3.4 | 0.4 | 92.6 | 4.3 | 176.7 | 5.2 |
| V1 | June 2015 | 4.0 | 0.5 | 105.8 | 6.9 | 112.8 | 4.7 |
| V2 | September 2016 | 1.3 | 0.3 | 55.8 | 3.5 | 139.9 | 6.2 |
| V3 | January 2016 | 6.5 | 1.3 | 139.1 | 12.7 | 244.9 | 15.1 |
| V4 | April 2016 | 2.8 | 0.4 | 78.6 | 4.5 | 150.3 | 5.8 |
| V5 | August 2016 | 3.3 | 1.0 | 106.0 | 12.0 | 279.7 | 13.1 |
| V6 | November 2016 | 4.7 | 0.6 | 171.2 | 7.7 | 406.2 | 11.3 |
| V7 | December 2016 | 3.1 | 0.6 | 167.2 | 7.2 | 328.7 | 11.6 |
| V8 | December 2016 | 4.5 | 0.8 | 135.9 | 8.3 | 261.9 | 12.6 |

| Sample | Type of sample | Al | Cr |  | Co |  | Ni |  | Cu |  | Zn |  | Sb |  | Pb |  | EF Pb | EF Cu | EF Zn | EF Ni | EF Cr | EF  Co | EF  Sb |
| --- | --- | --- | --- | --- | --- | --- | --- | --- | --- | --- | --- | --- | --- | --- | --- | --- | --- | --- | --- | --- | --- | --- | --- |
|  |  | **g.kg^-1^** | **mg.kg^-1^** | | | | | | | | | | | | | |  | | | | |  |  |
|  |  |  | **mean** | **SD** | **mean** | **SD** | **mean** | **SD** | **mean** | **SD** | **mean** | **SD** | **mean** | **SD** | **mean** | **SD** |  |  |  |  |  |  |  |
| A1 | Agricultural soil | 45.5 | 53.6 | -* | - | - | 35.2 | -* | 23.7 | -* | 63.1 | -* | - | - | 21.0 | -* | 1 | 2 | 1 | 1 | 1 | - | - |
| A2 | Agricultural soil | 38.3 | 49.6 | -* | - | - | 31.2 | -* | 33.6 | -* | 58.8 | -* | - | - | 27.6 | -* | 2 | 4 | 1 | 1 | 1 | - | - |
| A3 | Agricultural soil | 32.4 | 22.4 | -* | - | - | 21.3 | -* | 11.4 | -* | 31.9 | -* | - | - | 9.2 | -* | 1 | 2 | 1 | 1 | 1 | - | - |
| A4 | Agricultural soil | 13.5 | 10.8 | -* | - | - | 4.8 | -* | 8.4 | -* | 14.2 | -* | - | - | 2.3 | -* | 1 | 3 | 1 | 1 | 1 | - | - |
| A5 | Agricultural soil | 27.2 | 30.3 | 0.8 | 6.1 | 0.1 | 11.3 | 0.1 | 14.3 | 0.02 | 74.2 | 1.2 | 1.7 | 1.2 | 33.9 | 0.4 | 3 | 2 | 2 | 1 | 1 | 1 | 5 |
| RDS1 | RDS | 23.4 | 84.7 | 2.0 | 11.3 | 0.2 | 24.5 | 2.2 | 165.8 | 3.8 | 655.0 | 29.1 | 9.7 | 0.4 | 257.7 | 6.1 | 28 | 43 | 17 | 1 | 2 | 3 | 33 |
| RDS2 | RDS | 22.6 | 88.4 | 1.0 | 12.4 | 0.1 | 41.3 | 1.2 | 323.7 | 11.9 | 1274 | 85.3 | 32.5 | 0.9 | 188.1 | 2.1 | 22 | 59 | 39 | 2 | 2 | 3 | 115 |
| RDS3 | RDS | 25.4 | 94.1 | 4.2 | 12.9 | 0.7 | 40.2 | 2.8 | 259.6 | 8.5 | 914.3 | 4.0 | 22.2 | 1.5 | 185.1 | 11.0 | 16 | 32 | 22 | 2 | 2 | 3 | 70 |
| RDS4 | RDS | 25.5 | 89.0 | 2.7 | 14.5 | 0.6 | 43.8 | 1.8 | 407.0 | 1.3 | 935.2 | 25.3 | 23.0 | 0.6 | 216.6 | 10.7 | 13 | 33 | 15 | 2 | 1 | 3 | 72 |
| RB 1 | River bank | 22.5 | 24.1 | 0.5 | 5.3 | 0.2 | 13.2 | 0.4 | 16.2 | 0.6 | 73.3 | 3.5 | 1.2 | 0.1 | 91.4 | 4.0 | 11 | 3 | 2 | 1 | 0 | 1 | 4 |
| RB 2 | River bank | 34.8 | 39.0 | 1.9 | 7.2 | 0.1 | 17.9 | 1.3 | 12.9 | 0.4 | 62.5 | 1.2 | 0.8 | 0.02 | 19.4 | 0.6 | 2 | 2 | 1 | 1 | 0 | 1 | 2 |
| RB 3 | River bank | 26.2 | 32.6 | 1.2 | 5.6 | 0.2 | 14.3 | 0.6 | 17.4 | 0.7 | 105.6 | 3.3 | 2.7 | 3.1 | 34.5 | 1.4 | 4 | 3 | 3 | 1 | 0 | 1 | 8 |

Table S5. Trace elements concentrations, enrichment factors and aluminum content of agricultural soils, RDS and river bank samples; standard deviation (SD) obtained for duplicates ; -* SD not determined because of single determination

Table S6. Radionuclide activities in potential sediment sources collected in the Orge catchment (i.e. forest soils, agricultural soils, urban soils, road deposited sediments (RDS) and channel banks). <DL: lower than the detection limit (see Table S4).

|  | Type | Sampling date | ^137^Cs | SD (2σ) | ^210^Pb_xs_ | SD (2σ) | ^7^Be | SD (2σ) | Al |
| --- | --- | --- | --- | --- | --- | --- | --- | --- | --- |
|  |  |  | Bq.kg^-1^ | | | | | | g.kg^-1^ |
| F1 | Forest soil | 2015 | 19.0 | 0.4 | 17.2 | 1.5 | < DL | - | 13.8 |
| F2 | Forest soil | 2015 | 36.3 | 0.7 | 164.8 | 3.8 | < DL | - | 7.9 |
| F3 | Forest soil | 2015 | 11.9 | 0.3 | 66.7 | 2.0 | < DL | - | 5.2 |
| F4 | Forest soil | 2015 | 5.6 | 0.2 | 46.2 | 1.8 | < DL | - | 15.9 |
| F5 | Forest soil | 2015 | 4.5 | 0.3 | 5.7 | 2.3 | < DL | - | 23.1 |
| F6 | Forest soil | 2015 | 0.9 | 0.1 | 4.4 | 1.3 | < DL | - | 6.7 |
| F7 | Forest soil | 2015 | 13.7 | 0.5 | 49.9 | 3.3 | < DL | - | 6.01 |
| F8 | Forest soil | 2015 | 16.2 | 1.2 | 102.8 | 9.5 | < DL | - | 6.3 |
| F9 | Forest soil | 2017 | 5.2 | 0.2 | 14.9 | 1.9 | < DL | - | 15.2 |
| F10 | Forest soil | 2017 | 4.3 | 0.1 | 13.1 | 1.2 | < DL | - | 5.5 |
| A1 | Agricultural soil | 2015 | 3.6 | 0.2 | 0.0 | 3.4 | < DL | - | 45.5 |
| A2 | Agricultural soil | 2015 | 2.6 | 0.1 | 0.0 | 2.7 | < DL | - | 38.3 |
| A3 | Agricultural soil | 2015 | 2.2 | 0.1 | 0.0 | 1.5 | < DL | - | 32.4 |
| A4 | Agricultural soil | 2015 | 2.8 | 0.1 | 3.9 | 1.1 | < DL | - | 13.5 |
| A5 | Agricultural soil | 2017 | 4.7 | 0.2 | 0.0 | 3.0 | < DL | - | 27.2 |
| U1 | Urban soil | 2015 | 3.8 | 0.2 | 6.7 | 1.2 | < DL | - | 18.3 |
| U2 | Urban soil | 2015 | 1.8 | 0.1 | 0.0 | 2.7 | < DL | - | 20.3 |
| U3 | Urban soil | 2015 | 12.3 | 0.3 | 40.9 | 2.1 | < DL | - | 17.8 |
| U4 | Urban soil | 2015 | 2.8 | 0.1 | 4.3 | 0.8 | < DL | - | 19.4 |
| U5 | Urban soil | 2015 | 2.4 | 0.2 | 3.1 | 1.4 | < DL | - | 31.5 |
| U6 | Urban soil | 2015 | 12.6 | 0.4 | 17.0 | 3.2 | < DL | - | 30.8 |
| U7 | Urban soil | 2015 | 4.1 | 0.7 | 16.2 | 6.0 | < DL | - | 43.4 |
| U8 | Urban soil | 2015 | 4.4 | 0.2 | 0.0 | 4.0 | < DL | - | 11.8 |
| U9 | Urban soil | 2015 | 3.0 | 0.2 | 13.3 | 2.5 | < DL | - | 18.7 |
| U10 | Urban soil | 2015 | 1.3 | 0.2 | 0.0 | 5.3 | < DL | - | 28.2 |
| RDS1 | RDS | 2016 | 0.0 | 0.3 | 30.7 | 1.6 | 102.5 | 7.4 | 23.4 |
| RDS2 | RDS | 2016 | 1.2 | 0.4 | 387.2 | 8.0 | 704.1 | 39.7 | 22.6 |
| RDS3 | RDS | 2016 | 1.2 | 0.3 | 296.3 | 6.8 | 412.3 | 29.8 | 25.4 |
| RDS4 | RDS | 2016 | 0.7 | 0.3 | 168.4 | 5.2 | 322.6 | 26.0 | 25.5 |
| RB 1 | River bank | 2016 | 3.8 | 0.2 | 7.6 | 1.2 | 0.0 | 1.9 | 22.5 |
| RB 2 | River bank | 2016 | 1.3 | 0.1 | 11.8 | 1.0 | 0.0 | 1.9 | 34.8 |
| RB 3 | River bank | 2016 | 0.2 | 0.1 | 8.4 | 1.2 | 3.1 | 1.2 | 26.2 |

Table S7. Results of the calculated sediment contributions using the mean and highest/lowest radionuclide activities measured in road deposited sediments (RDS) for resolving the two-end-member equation (see the section 2.5)

|  | Contribution calculations | | | | | | | | | |
| --- | --- | --- | --- | --- | --- | --- | --- | --- | --- | --- |
|  | Using median RDS ^7^Be and ^210^Pb_xs_ | | Using high RDS ^7^Be and ^210^Pb_xs_ | | Using low RDS ^7^Be and ^210^Pb_xs_ | | **Mean contributions** | | | |
|  | Old particles | RDS contribution | Old particles | RDS contribution | Old particles | RDS contribution | Old particle | | RDS contribution | |
|  | Contribution in SPM in % | | Contribution in SPM in % | | Contribution in SPM in % | | Mean | SD | Mean | SD |
| D1 | 95 | 5 | 97 | 3 | 94 | 6 | 95 | 2 | 5 | 2 |
| D2 | 87 | 13 | 92 | 8 | 83 | 17 | 87 | 5 | 13 | 5 |
| D3 | 88 | 12 | 93 | 7 | 85 | 15 | 89 | 4 | 11 | 4 |
| D4 | 87 | 13 | 92 | 8 | 83 | 17 | 88 | 5 | 12 | 5 |
| D5 | 97 | 3 | 98 | 2 | 96 | 4 | 97 | 1 | 3 | 1 |
| D6 | 87 | 13 | 92 | 8 | 84 | 16 | 88 | 4 | 12 | 4 |
| D7 | 89 | 11 | 93 | 7 | 86 | 14 | 89 | 4 | 11 | 4 |
| D8 | 86 | 14 | 92 | 8 | 82 | 18 | 87 | 5 | 13 | 5 |
| E1 | 89 | 11 | 93 | 7 | 86 | 14 | 89 | 4 | 11 | 4 |
| E2 | 64 | 36 | 78 | 22 | 50 | 50 | 64 | 14 | 36 | 14 |
| E3 | 89 | 11 | 93 | 7 | 85 | 15 | 89 | 4 | 11 | 4 |
| E4 | 75 | 25 | 85 | 15 | 68 | 32 | 76 | 9 | 24 | 9 |
| E5 | 88 | 12 | 93 | 7 | 84 | 16 | 88 | 4 | 12 | 4 |
| E6 | 80 | 20 | 88 | 12 | 75 | 25 | 81 | 7 | 19 | 7 |
| Y1 | 72 | 28 | 83 | 17 | 61 | 39 | 72 | 11 | 28 | 11 |
| Y2 | 64 | 36 | 78 | 22 | 48 | 52 | 63 | 15 | 37 | 15 |
| Y3 | 67 | 33 | 80 | 20 | 57 | 43 | 68 | 12 | 32 | 12 |
| Y4 | 70 | 30 | 81 | 19 | 57 | 43 | 69 | 12 | 31 | 12 |
| Y5 | 71 | 29 | 83 | 17 | 63 | 37 | 72 | 10 | 28 | 10 |
| Y6 | 61 | 39 | 76 | 24 | 45 | 55 | 61 | 16 | 39 | 16 |
| V1 | 73 | 27 | 84 | 16 | 65 | 35 | 74 | 10 | 26 | 10 |
| V2 | 77 | 23 | 86 | 14 | 67 | 33 | 77 | 9 | 23 | 9 |
| V3 | 41 | 59 | 65 | 35 | 24 | 76 | 43 | 21 | 57 | 21 |
| V4 | 67 | 33 | 80 | 20 | 53 | 47 | 67 | 13 | 33 | 13 |
| V5 | 55 | 45 | 73 | 27 | 37 | 63 | 55 | 18 | 45 | 18 |
| V6 | 27 | 73 | 56 | 44 | -* | -* | 41 | 21 | 59 | 21 |
| V7 | 29 | 71 | 57 | 43 | 1 | 99 | 29 | 28 | 71 | 28 |
| V8 | 42 | 58 | 65 | 35 | 19 | 81 | 42 | 23 | 58 | 23 |
| *: sample with radionuclide activities higher than those found in the potential sources | | | | | | | | | | |

Table S8. Contribution of recently eroded particles estimated for the SPM samples collected in the Orge River based on the ^7^Be/^210^Pb_xs_ ratios model (Matisoff et al., 2005)

|  | Campaign | Contribution of recently eroded particles (%) |
| --- | --- | --- |
| D1 | June 2015 | 26 |
| D2 | September 2015 | 39 |
| D3 | January 2016 | 30 |
| D4 | April 2016 | 34 |
| D5 | August 2016 | 16 |
| D6 | November 2016 | 33 |
| D7 | December 2016 | 36 |
| D8 | December 2016 | 39 |
| E1 | June 2015 | 26 |
| E2 | September 2015 | 34 |
| E3 | January 2016 | 66 |
| E4 | April 2016 | 24 |
| E5 | August 2016 | 54 |
| E6 | November 2016 | 37 |
| Y1 | June 2015 | 49 |
| Y2 | September 2015 | 64 |
| Y3 | January 2016 | 66 |
| Y4 | April 2016 | 48 |
| Y5 | August 2016 | 64 |
| Y6 | November 2016 | 51 |
| V1 | June 2015 | 57 |
| V2 | September 2015 | 32 |
| V3 | January 2016 | 75 |
| V4 | April 2016 | 53 |
| V5 | August 2016 | 57 |
| V6 | November 2016 | 79 |
| V7 | December 2016 | 71 |
| V8 | December 2016 | 59 |

Table S9. SPM concentration at each sampling site and for each campaign

|  | Campaign | SPM concentration (mg.L^-1^) |
| --- | --- | --- |
| D1 | June 2015 | 117.0 |
| D2 | September 2015 | 30.2 |
| D3 | January 2016 | 33.2 |
| D4 | April 2016 | 10.7 |
| D5 | August 2016 | 34.4 |
| D6 | November 2016 | 6.8 |
| D7 | December 2016 | 6.8 |
| E1 | June 215 | 83.5 |
| E2 | September 2015 | 42.0 |
| E3 | January 2016 | 13.1 |
| E4 | April 2016 | 11.7 |
| E5 | August 2016 | 17.4 |
| E6 | November 2016 | 7.1 |
| Y1 | June 2015 | 76.0 |
| Y2 | September 2015 | 143.9 |
| Y3 | January 2016 | 13.4 |
| Y4 | April 2016 | 14.1 |
| Y5 | August 2016 | 22.8 |
| Y6 | November 2016 | 10.6 |
| V1 | June 2015 | 8.5 |
| V2 | September 2015 | 49.0 |
| V3 | January 2016 | 21.8 |
| V4 | April 2016 | 10.2 |
| V5 | August 2016 | 7.0 |
| V6 | November 2016 | 11.0 |
| V7 | December 2016 | 11.0 |

|  |  | Pb | Cu | Zn | Sb | ^206^Pb/^207^Pb | | ^208^Pb/^206^Pb | |
| --- | --- | --- | --- | --- | --- | --- | --- | --- | --- |
|  |  | mg.kg^-1^ | | | |  | **^2s^** |  | ^2s^ |
| This study | RDS1 | 234  ±52 | 228  ±144 | 581  ±63 | 6.9  ±0.9 | 1.1591 | 0.0019 | 2.1069 | 0.0044 |
|  | RDS2 | 183  ±29 | 307  ±16 | 1322  ±31 | 30.6  ±0.8 | 1.1521 | 0.0018 | 2.1132 | 0.0018 |
|  | RDS3 | 147  ±10 | 186  ±11 | 825  ±99 | 19.9  ±2.6 | 1.1492 | 0.0012 | 2.1146 | 0.0020 |
|  | RDS4 | 122  ±17 | 191  ±11 | 551  ±33 | 12.8  ±1.1 | 1.1512 | 0.0006 | 2.1146 | 0.0011 |
|  | RDS Dourdan 1 | 34.5  ±13.1 | 138.6  ±78.7 | 195.8  ±13.9 | 6.1  ±1.2 | 1.1613 | 0.0029 | 2.1105 | 0.0145 |
|  | RDS Dourdan 2 | 32.8  ±16.7 | 58.5  ±17.1 | 122.4  ±21.4 | 3.3  ±0.6 | 1.1576 | 0.0039 | 2.1057 | 0.0032 |
| *Manchester, UK^a^* | City (2001) | 354 | 92 | 706 | - | - |  | - |  |
|  | Highway (2001) | 185 | 132 | 606 | - | - |  | - |  |
| *Gold Coast, Australia^b^* | Clearview Estate | 32.5 | 131.4 | 296.6 | - | - |  | - |  |
|  | Nerang | 25.7 | 65.5 | 176.4 | - | - |  | - |  |
|  | Benowa | 29.1 | 98.4 | 236.5 | - | - |  | - |  |
|  | Surfers Paradise | 38.4 | 70.8 | 90.4 | - | - |  | - |  |
| *Japan^c^* | Tokyo | 245.0 | - | 1888.0 | - | 1.145 |  | 2.124 |  |
|  | Osaka | 156.5 | - | 934.5 | - | 1.147 |  | 2.117 |  |
|  | Kyoto | 72.6 | - | 1170 | - | 1.152 |  | 2.115 |  |
| *Bangkok, Thailand^d^* | Mo Chit | 65.8 | - | 872 | - | 1.147 |  | 2.116 |  |
|  | Victory Monument | 38.1 | - | 414 | - | 1.144 |  | 2.115 |  |
|  | Siam Square | 63.8 | - | 526 | - | 1.153 |  | 2.108 |  |
|  | Taksin | 150 | - | 2571 | - | 1.145 |  | 2.118 |  |

Table S10. Median concentrations of TE and lead isotopic ratios in road deposited sediments (RDS) and comparison with the literature

^a^: (Robertson et al., 2003); ^b^: (Gunawardana et al., 2012); ^c^: (Wijaya et al., 2012); ^d^: (Wijaya et al., 2013)

Table S10. Correlation matrix of trace elements in suspended particulate matter SPM collected in the Orge River (France) from June 2015 to December 2016.

|  | V | Cr | Mn | Co | Ni | Fe | Cu | Zn | As | Se | Rb | Sr | Mo | Ag | Cd | Sb | Cs | Ba | Tl |
| --- | --- | --- | --- | --- | --- | --- | --- | --- | --- | --- | --- | --- | --- | --- | --- | --- | --- | --- | --- |
| V | 1.0 |  |  |  |  |  |  |  |  |  |  |  |  |  |  |  |  |  |  |
| Cr | 0.9 | 1 |  |  |  |  |  |  |  |  |  |  |  |  |  |  |  |  |  |
| Mn | 0.9 | 0.9 | 1.0 |  |  |  |  |  |  |  |  |  |  |  |  |  |  |  |  |
| Co | 0.9 | 0.7 | 0.8 | 1.0 |  |  |  |  |  |  |  |  |  |  |  |  |  |  |  |
| Ni | 1.0 | 0.9 | 0.9 | 0.9 | 1.0 |  |  |  |  |  |  |  |  |  |  |  |  |  |  |
| Fe | 0.9 | 0.9 | 0.9 | 0.9 | 0.9 | 1.0 |  |  |  |  |  |  |  |  |  |  |  |  |  |
| Cu | 0.5 | 0.7 | 0.5 | 0.1 | 0.3 | 0.4 | 1.0 |  |  |  |  |  |  |  |  |  |  |  |  |
| Zn | 0.5 | 0.7 | 0.5 | 0.1 | 0.3 | 0.3 | 1.0 | 1.0 |  |  |  |  |  |  |  |  |  |  |  |
| As | 0.8 | 0.7 | 0.7 | 0.9 | 0.9 | 0.8 | 0.1 | 0.1 | 1.0 |  |  |  |  |  |  |  |  |  |  |
| Se | 0.1 | 0.1 | 0.1 | 0.3 | 0.1 | 0.3 | 0.0 | 0.0 | -0.2 | 1.0 |  |  |  |  |  |  |  |  |  |
| Rb | 1.0 | 0.9 | 0.9 | 0.8 | 0.9 | 0.9 | 0.4 | 0.5 | 0.8 | 0.0 | 1.0 |  |  |  |  |  |  |  |  |
| Sr | 0.5 | 0.7 | 0.6 | 0.2 | 0.4 | 0.4 | 0.9 | 0.9 | 0.2 | -0.1 | 0.5 | 1.0 |  |  |  |  |  |  |  |
| Mo | -0.5 | -0.4 | -0.5 | -0.6 | -0.5 | -0.6 | 0.1 | 0.1 | -0.3 | -0.4 | -0.5 | -0.1 | 1.0 |  |  |  |  |  |  |
| Ag | 0.4 | 0.5 | 0.3 | 0.1 | 0.2 | 0.3 | 0.9 | 0.8 | 0.0 | 0.2 | 0.3 | 0.8 | 0.1 | 1.0 |  |  |  |  |  |
| Cd | -0.4 | -0.3 | -0.5 | -0.3 | -0.4 | -0.3 | 0.1 | 0.1 | -0.5 | 0.6 | -0.4 | -0.1 | 0.3 | 0.2 | 1.0 |  |  |  |  |
| Sb | 0.3 | 0.6 | 0.3 | 0.0 | 0.2 | 0.3 | 0.9 | 0.9 | 0.0 | 0.1 | 0.3 | 0.8 | 0.2 | 0.9 | 0.2 | 1.0 |  |  |  |
| Cs | 1.0 | 0.9 | 0.9 | 0.9 | 0.9 | 0.9 | 0.4 | 0.4 | 0.8 | 0.1 | 1.0 | 0.5 | -0.6 | 0.4 | -0.4 | 0.3 | 1.0 |  |  |
| Ba | 0.8 | 0.8 | 0.8 | 0.5 | 0.7 | 0.6 | 0.6 | 0.6 | 0.6 | -0.3 | 0.8 | 0.8 | -0.4 | 0.5 | -0.6 | 0.4 | 0.8 | 1.0 |  |
| Tl | 0.6 | 0.4 | 0.5 | 0.8 | 0.6 | 0.7 | -0.2 | -0.2 | 0.6 | 0.5 | 0.6 | -0.2 | -0.5 | -0.2 | 0.0 | -0.2 | 0.6 | 0.0 | 1.0 |

Figure S1. Concentrations in K, Na and Mg (in g.kg^-1^) measured in the Orge River SPM and in potential sediment sources (i.e. river banks, RDS, agricultural, urban and forest soils)


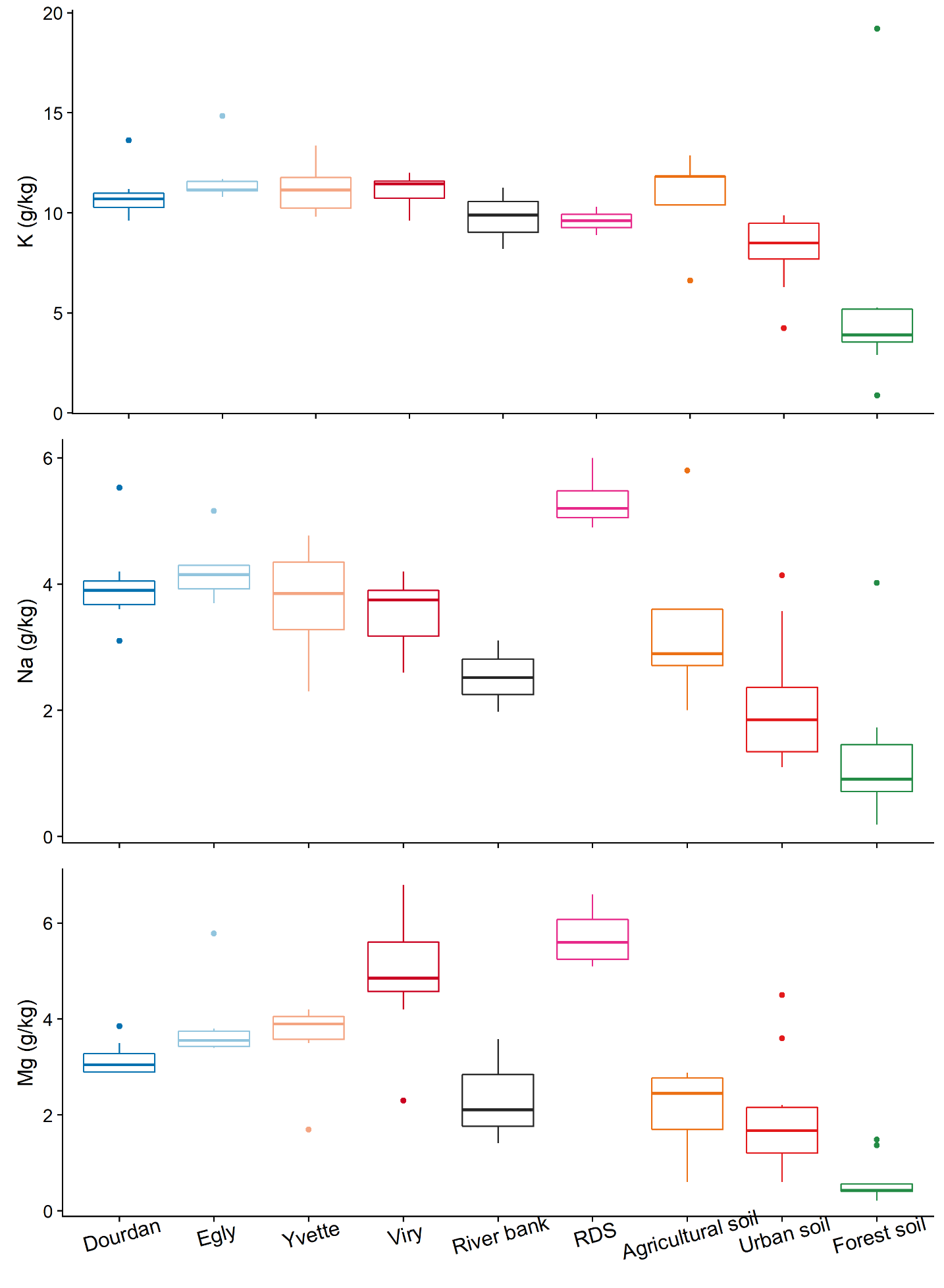


References

Gunawardana, C., Goonetilleke, A., Egodawatta, P., Dawes, L., Kokot, S., 2012. Source characterisation of road dust based on chemical and mineralogical composition. Chemosphere 87, 163–170. https://doi.org/10.1016/j.chemosphere.2011.12.012

Matisoff, G., Wilson, C.G., Whiting, P.J., 2005. The 7Be/210Pbxs ratio as an indicator of suspended sediment age or fraction new sediment in suspension. Earth Surf. Process. Landforms 30, 1191–1201. https://doi.org/10.1002/esp.1270

Robertson, D.J., Taylor, K.G., Hoon, S.R., 2003. Geochemical and mineral magnetic characterisation of urban sediment particulates, Manchester, UK. Appl. Geochemistry 18, 269–282. https://doi.org/10.1016/S0883-2927(02)00125-7

Wijaya, A.R., Ouchi, A.K., Tanaka, K., Cohen, M.D., Sirirattanachai, S., Shinjo, R., Ohde, S., 2013. Evaluation of heavy metal contents and Pb isotopic compositions in the Chao Phraya River sediments: Implication for anthropogenic inputs from urbanized areas, Bangkok. J. Geochemical Explor. 126–127, 45–54. https://doi.org/10.1016/j.gexplo.2012.12.009

Wijaya, A.R., Ouchi, A.K., Tanaka, K., Shinjo, R., Ohde, S., 2012. Metal contents and Pb isotopes in road-side dust and sediment of Japan. J. Geochemical Explor. 118, 68–76. https://doi.org/10.1016/j.gexplo.2012.04.009
